# Heatwave frequency and seedling death alter stress-specific emissions of volatile organic compounds in Aleppo pine

**DOI:** 10.1007/s00442-021-04905-y

**Published:** 2021-04-09

**Authors:** Benjamin Birami, Ines Bamberger, Andrea Ghirardo, Rüdiger Grote, Almut Arneth, Elizabeth Gaona-Colmán, Daniel Nadal-Sala, Nadine K. Ruehr

**Affiliations:** 1 Karlsruhe Institute of Technology KIT, Institute of Meteorology and Climate Research–Atmospheric Environmental Research, 82467 Garmisch-Partenkirchen, Germany; 2 University of Bayreuth, Bayreuth Center of Ecology and Environmental Research (BayCEER), Atmospheric Chemistry, Dr.-Hans-Frisch-Straße 1-3, 95448 Bayreuth, Germany; 3 Research Unit Environmental Simulation, Institute of Biochemical Plant Pathology, Helmholtz Zentrum München, Ingolstädter Landstr. 1, 85764 Neuherberg, Germany; 4 University of Bayreuth, Chair of Plant Ecology, Universitätsstraße 30, 95440 Bayreuth, Germany

**Keywords:** BVOC emissions, Drought, Heatwaves, Mortality, Stress-specific

## Abstract

Biogenic volatile organic compounds (BVOC) play important roles in plant stress responses and can serve as stress indicators. While the impacts of gradual environmental changes on BVOCs have been studied extensively, insights in emission responses to repeated stress and recovery are widely absent. Therefore, we studied the dynamics of shoot gas exchange and BVOC emissions in *Pinus halepensis* seedlings during an induced moderate drought, two four-day-long heatwaves, and the combination of drought and heatwaves. We found clear stress-specific responses of BVOC emissions. Reductions in acetone emissions with declining soil water content and transpiration stood out as a clear drought indicator. All other measured BVOC emissions responded exponentially to rising temperatures during heat stress (maximum of 43 °C), but monoterpenes and methyl salicylate showed a reduced temperature sensitivity during the second heatwave. We found that these decreases in monoterpene emissions between heatwaves were not reflected by similar declines in their internal storage pools. Because stress intensity was extremely severe, most of the seedlings in the heat-drought treatment died at the end of the second heatwave (dark respiration ceased). Interestingly, BVOC emissions (methanol, monoterpenes, methyl salicylate, and acetaldehyde) differed between dying and surviving seedlings, already well before indications of a reduced vitality became visible in gas exchange dynamics. In summary, we could clearly show that the dynamics of BVOC emissions are sensitive to stress type, stress frequency, and stress severity. Moreover, we found indications that stress-induced seedling mortality was preceded by altered methanol, monoterpene, and acetaldehyde emission dynamics.

**Supplementary Information:**

The online version contains supplementary material available at 10.1007/s00442-021-04905-y.

## Introduction

Climate change is expected to cause not only higher temperatures and a higher variability of precipitation, but also to produce more frequent and more intense extreme events such as heatwaves and drought spells (Baldwin et al. 2019; Kornhuber et al. 2019). This is likely to intensify forest degradation as has been already observed in many areas world-wide (Anderegg et al. 2019; Brodribb et al. 2020; Hartmann et al. 2018a). In particular, the co-occurrences of high temperatures and low water availability seems to damage tree growth and trigger mortality (Choat et al. 2018; Hartmann et al. 2018b; Ruehr et al. 2019; Williams et al. 2013). Nevertheless, the specific mechanisms of this phenomenon are still heavily discussed, since it is unclear how drought and heat effects interact and when stress-induced mortality actually occurs (Hammond et al. 2019; Hartmann et al. 2018b).

At the onset of severe droughts, trees initially react by closing their stomata to prevent excessive water loss, which in turn leads to suboptimal leaf internal carbon dioxide concentrations (*C*_i_) and eventually limits photosynthesis (*A*_net_) (Brunner et al. 2015; Gupta et al. 2020). As drought intensifies, the water potential of the conductive xylem can drop below a species-specific critical threshold (Anderegg et al. 2019; Ruehr et al. 2019), followed by embolism impairing water transport. At this point, the probability of drought-induced mortality increases (Hammond et al. 2019) because living tissue becomes dehydrated (Körner 2019). If the stress is not lethal, the organism requires carbon for repair and/or recovery processes, which is why individuals might still die sometime after the stress ceased, if sufficient reserves are not available (Ruehr et al. 2019).

High temperatures have the potential to increase physiological drought stress by increasing the vapor pressure deficit (VPD) of the surrounding air, which then leads to an increase in water loss by transpiration (*E*) (Panek and Goldstein 2001). Heat stress is amplified by limited water availability because reduced evaporation limits the possibility for cooling the leaf surface (Ruehr et al. 2016; Williams et al. 2013). High temperatures will first speed up biochemical reactions, reducing the lifetime of proteins and causing imbalances primarily in the energy-providing pathways (light assimilation, photosynthesis, respiration) (Niinemets 2018). Apart from higher resource requirements, this response enhances the formation of harmful reactive oxidative species (ROS) (Escandón et al. 2016; Song et al. 2014). Finally, a very high temperature may well lead to direct membrane damages, induce necrosis and eventually tissue senescence (Colombo and Timmer 1992; Daniell et al. 1969; Hüve et al. 2011) and can also lead to mortality (Birami et al. 2018).

Apart from opening stomata to increase evaporative cooling, which increases the risk of dehydration, the production of biogenic volatile organic compounds (BVOC) is another response to cope with abiotic stress (Spinelli et al. 2011). In particular terpenoids such as isoprene, monoterpenes (MT), and sesquiterpenes (SQT) play important roles in detoxifying reactive substances, regardless if these are taken up or formed internally in response to heat or radiation (Nogués et al. 2015; Vickers et al. 2009). A second protective mechanism is the stabilization of membranes that is established by the incorporation of isoprene or MT (Loreto et al. 1998; Mahajan et al. 2019). In addition, it seems that isoprenoids could also act as signaling molecules inducing a network of transcription factors that may play a role for stress tolerance (Harvey and Sharkey 2016).

Under stress conditions, new BVOCs may be emitted, or constitutively emitted BVOCs may increase several-folds above their unstressed rates (Guidolotti et al. 2019; Joó et al. 2011; Yáñez-Serrano et al. 2019). Such stress-induced BVOC emissions can either originate from de novo biosynthesis or are previously formed compounds, which had been stored in specific structures (e.g. MT from resin ducts in coniferous species) (Ghirardo et al. 2010; Turan et al. 2019). Typically damage-released compounds besides isoprene, mono- and sesquiterpenes, are green leaf volatiles, methanol and acetaldehyde as well as methyl salicylate (MeSa) (Guidolotti et al. 2019; Joó et al. 2011).

Thus, with the ongoing rise of temperatures as well as increased frequency and intensity of heatwaves and drought spells, changes in BVOC emissions can be expected. While most volatile emissions have been found to generally increase with temperature (Niinemets et al. 2010), this effect is less clear in response to drought and emissions patterns differ with species and drought intensity. Some authors found that emissions are increased at mild drought stress, while a chronic, prolonged drought decreases emissions (Dani et al. 2015; Eller et al. 2016; Llusia et al. 2016). However, significant amounts of MT emissions were still found in Aleppo pines at very dry conditions where photosynthesis was already dramatically reduced (Seco et al. 2017). So, not only the intensity but also the composition of emissions is likely to change, particularly under extreme events, which would influence vegetation-climate interactions (Harper and Unger 2018; Sporre et al. 2019). BVOCs take part in air chemistry processes and affect regional ozone concentration as well as aerosol abundance, with secondary impacts on cloud formation and radiation balance (Porter and Heald 2019; Zhao et al. 2017). Globally, BVOCs reduce the abundance of radicals in the air and thus increase the longevity of greenhouse gases, i.e. methane (Fuentes et al. 2001; Monson and Holland 2001). Hence, elucidating the variety of BVOC emission responses to different intensity, elongation, and frequency of stress conditions is needed.

Monoterpenes can be found in most conifers, becoming a main compound of resin, stored in large amounts in specialized resin ducts (Celedon and Bohlmann 2019; Turner et al. 2019). Despite representing large storage pools (up to 0.8% of the dry needle matter, (Vanhatalo et al. 2018)), it has been found that in Scots pine 10–58% of the emitted MT can still be synthesized de novo from freshly fixed atmospheric carbon (Ghirardo et al. 2010; Kleist et al. 2012). Similarly, about half of MT measured in Aleppo pine in spring were estimated to be light-dependent (Llusia et al. 2016). Apart from specialized storages, MT can be stored non-specifically as glycosylates in micro vesicles, or be integrated with biological membranes (Nagegowda 2010; Yazaki et al. 2017), and even accumulate in epicuticular wax layers (Joensuu et al. 2016). From these unspecific storages, MT can be directly released. Apart from MT, typically stress-induced terpenoids are sesquiterpenes (SQT), which play a role in plant-to-plant signaling in tree defense strategies against insects and pathogens (Joó et al. 2011; Kleist et al. 2012).

In addition to terpenoid emissions, a relatively large amount of BVOCs are oxygenated compounds that originate from various biochemical pathways (Grote et al. 2019). The most abundant in the atmosphere is methanol (Jacob 2005), which is formed mainly during cell wall development and can act as a stress signal transmitter (Dorokhov et al. 2018). Other short-chain volatile organic compounds (VOC) are derived downstream of glycolysis from either pyruvate or acetyl-CoA (Fall 2003; Grote et al. 2019), during anoxic stress conditions (Kelsey et al. 2011; Kreuzwieser et al. 1999), indicating substrate overflow mechanisms (Karl et al. 2002). Thus, acetaldehyde, ethanol, and acetone are often produced in roots, phloem or cambial tissues (Kimmerer and Stringer 1988; Rissanen et al. 2020) where they remain dissolved until they reach the leaves via the transpiration stream within the plant (Rissanen et al. 2018). Some of them, especially methanol and acetaldehyde are indicators of high metabolic activity, often found when tissue damage occurs (Fall et al. 1999; Kreuzwieser et al. 1999; Loreto et al. 2006; Portillo-Estrada et al. 2015; Turan et al. 2019).

Dependencies of constitutively emitted BVOCs on temperature have been described extensively (Grote et al. 2014; Guenther et al. 1993; Niinemets et al. 2002), while deficits still exist in representing stress-induced BVOC emissions. Particularly, the emission responses to repeated heat stress under well-watered or drought-exposed conditions have not received much attention so far. BVOC emission might be affected by potential acclimation responses and the production of compounds might be limited by decreasing carbon supply (Jud et al. 2016; Vanzo et al. 2015). In the case of storage-released terpenoids, the storage capacity might decline and limit emissions during repeated and/or long-term heat stress (Schurgers et al. 2009). Finally, it remains unknown if BVOC emissions of coniferous trees prone to death differ from surviving trees, and hence may provide a death-preceding indicator of mortality.

To investigate BVOC responses to heat and combined heat-drought stress, we selected Aleppo pine, a tree species common to the dry and semi-arid regions in the Mediterranean area (Mauri et al. 2016). The seedlings used here originate from the Yatir forest in Israel, an Aleppo pine plantation at the border to the Negev desert. Based on the literature, we hypothesize that (1) Aleppo pine will change BVOC emissions quantitatively and qualitatively under a combined heat and drought stress and that this response differs from that to only one of these stresses. (2) Emission bouquets differ between first and repeated stress caused by a reduction of storage compounds. (3) If stress induces seedling mortality, the emission is different from those of surviving seedlings.

## Materials and methods

### Plant cultivation

**Fig. 1:**
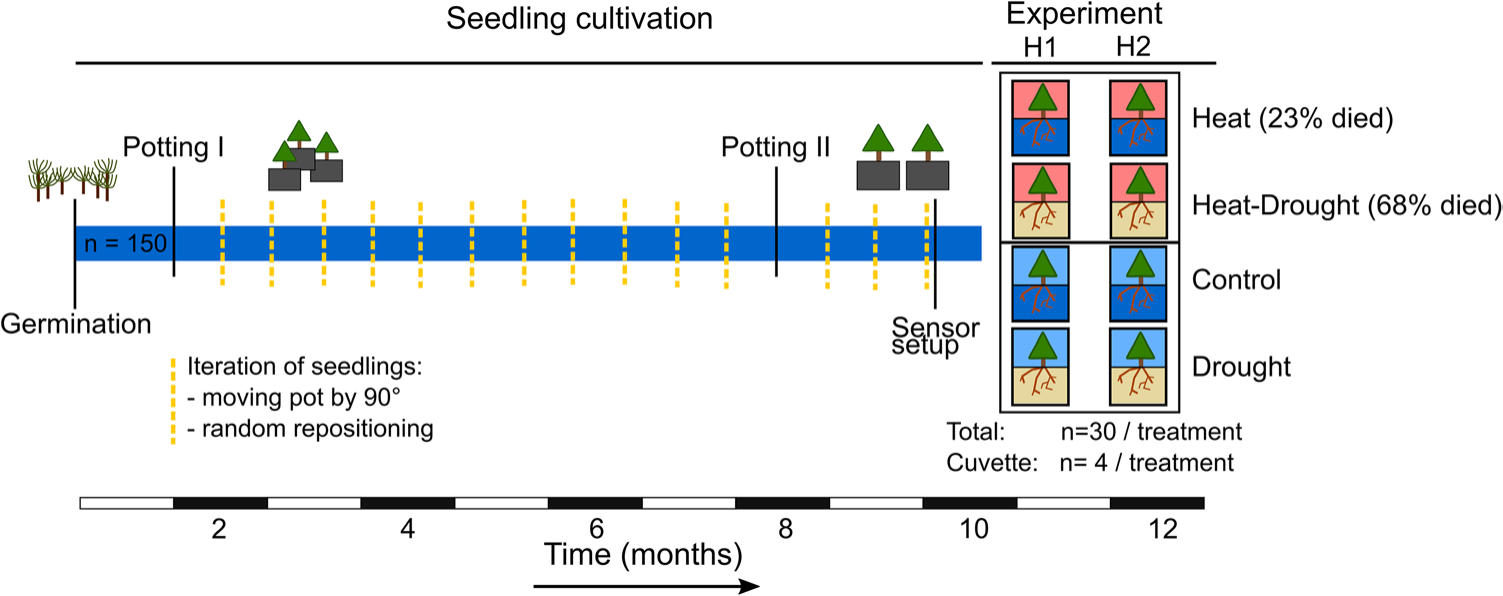
Experimental timeline: from Aleppo pine seedling germination until the drought was initiated and two heat experiments (each 4 d) were conducted 10 months later. During the cultivation phase, all seedlings were grown in one of the climate-controlled greenhouse compartments and positioning among seedlings was regularly iterated. Before the stress experiment was initiated the seedlings were randomly placed in two separate greenhouse compartments to conduct heatwave scenarios. In each treatment, four seedlings were placed in light-transmitting gas exchange cuvettes coated with Teflon on the inside. In the heat and heat-drought treatment seedlings died due to overheating during the course of the experiment, mostly during the short recovery phase between heatwave 1 (H1) and heatwave 2 (H2) (see Birami et al. 2018 for details). Note that also seedlings placed in the gas exchange cuvettes died: one seedling in the heat treatment and three seedlings in the heat-drought treatment

Aleppo pine (*Pinus halepensis*, Miller) seedlings were cultivated from seeds in a controlled greenhouse environment in Garmisch-Partenkirchen, Germany (732 m a.s.l., 47°28′32.87′’N, 11°3′44.03′’E). Seed material originated from five different trees in a 55-year-old Aleppo pine plantation in Israel (Yatir forest, IL-Yat, 650 m a.s.l., 31°20′49.2′’N, 35°03′07.2′’E). The genetic heritage is not clear (Schiller and Atzmon 2009), but due to high levels of heterozygosity, seedling offspring may be referred to as a mixed F1 population (Atzmon et al. 2004; Korol and Schiller 1996). Seedlings (4 weeks old, July 2015) were transplanted to 1 L pots containing mineral substrate (2:8) of quartz sand (0.7 mm and 1–2 mm) and vermiculite (ca. 3 mm) with 2 g of slow-release fertilizer (Osmocote® Exact + TE 3–4 months fertilizer 16–9–12 + 2MgO + TE, Everis International B.V., Heerlen, The Netherlands). Seven-month-old seedlings (January 2016) were planted in larger pots (2.5 L) containing mineral substrate (1:2:1) of quartz sand (1–2 mm): quartz sand (4–6 mm): vermiculite (3 mm) and 6 g of slow-release fertilizer (Osmocote® Exact + TE 5–6 month fertilizer 15–9–12 + 2MgO + TE). Air temperature and relative humidity (RH) during cultivation (Table S1) were set to mimic the monthly mean of temperature and humidity data of the Yatir forest (averaged over ten years) as described previously (Birami et al. 2018). The position of the seedlings within the greenhouse compartment was iterated randomly to avoid any positioning effect (Fig. 1). Nine-month-old seedlings were divided into four treatments in March 2016: control, heat, drought, and heat-drought one month before initiating the heatwave experiment (experiment in April 2016, Fig. 1). Therefore, 30 seedlings each of the control and drought treatments, as well as the heat and heat-drought treatments were placed into two adjacent environmentally-controlled compartments using a randomized block design (Birami et al. 2018; Ruehr et al. 2016). Substrate-specific calibrated moisture sensors (10HS, Decagon Devices, Inc., WA, USA) have been installed (*n* = 10 per treatment). Per treatment, four seedlings each were equipped with a gas exchange cuvette (*n* = 4 per treatment; see method Sect. 2.3 for details). Therefore, iteration of the seedlings’ placement during the experimental phase was no longer possible.

### Experimental setup

Seedlings of the heat and heat-drought treatment were exposed to two heatwaves with stepwise increasing temperatures and vapor pressure deficit (VPD) (Fig. S1). The heatwaves were designed to mimic naturally occurring few day-long heatwaves in the Yatir forest typically occurring during early summer (Tatarinov et al. 2016). In our experiment, each heatwave had a duration of 4 days (April 27th–April 30th and May 7th–May 10th, 2016) and temperature was increased during the first three days. The temperature level of the third day was repeated on the fourth day with a maximum of 42.8 °C during the first heatwave (H1) and 42.2 °C during the second heatwave (H2; see Table 1; Fig. S1). The trajectories of two heatwaves differed slightly, with the second heatwave reaching temperatures > 40 °C already at day two (compared to day three during H1). Note that the seedlings enclosed in cuvettes for BVOC and gas exchange measurements (see Sect. 2.3, Fig. S2), experienced higher temperatures after the lid of the cuvettes closed automatically (on average 3.8 ± 1 °C at the end of the 10 min measurement cycle; 3-times per day). The average light intensity was 416 ± 105 µmol m^−2^ s^−1^ during day-time and water vapor in the greenhouse compartments and gas exchange cuvettes was kept constant, which resulted in 20–40% RH and an increase of VPD to a maximum of 7.5 kPa during the heatwaves in the greenhouse compartment, similar to VPD conditions at the Yatir forest during heatwave periods in early summer (Tatarinov et al. 2016). The 1-month drought period was initiated (DOY 114) 4 days before the first heatwave (DOY 118–121) and ended (DOY 138) 7 days after the second heatwave (DOY 128–131). Irrigation was reduced to a relative substrate water content (rSWC) of about 15% in the drought and heat-drought treatment, while it was kept between 40 and 50% under well-watered and control conditions (Fig. S1).

**Table 1:** Air temperature (T_Air_) and vapor pressure deficit (VPD) before, during the two heatwaves and after. Averages (± 1SD) and minima and maxima are given for each day of the heatwaves and for the periods before, between and after the heatwaves. Note minima occurred during night-time (8 h), maxima during day-time (16 h)

		*T*_Air_ [°C]			VPD [kPa]		
		Min	Mean	Max	Min	Mean	Max
	Before	11.1	18.3 ± 2.7	24.8	0.2	1.1 ± 0.4	2.3
Heat-wave I	Day 1	14.4	21.3 ± 3.9	27.1	0.5	1.7 ± 0.7	2.9
	Day 2	17.7	28.2 ± 4.5	33.7	1.1	2.8 ± 1	4.3
	Day 3	25.2	33.5 ± 5	42.8	1.8	4.1 ± 1.7	7.4
	Day 4	17.9	33.5 ± 7	42.2	1.2	4.2 ± 1.9	7.1
	Between	15.7	20.7 ± 3	27	0.6	1.4 ± 0.5	2.6
Heat-wave II	Day1	17.0	26 ± 4.8	32.1	0.9	2.4 ± 1	3.9
	Day2	18.6	31.4 ± 6.5	40.2	1.1	3.7 ± 1.7	6.4
	Day3	25.8	34 ± 5.4	41.5	1.9	4.2 ± 1.7	6.9
	Day4	17.8	33.4 ± 6.7	42.2	0.9	3.9 ± 1.8	6.9
	After	16.7	21.5 ± 2.9	26.5	0.6	1.4 ± 0.5	2.5

### Gas exchange and BVOC emission analyses

A custom-made, open chamber system, which has been previously described (Bamberger et al. 2017; Birami et al. 2018; Duarte et al. 2016), was used to automatically measure gas exchange and BVOC emissions from the shoots of the seedlings as follows. Randomly selected seedlings (*n* = 4 per treatment) were distributed spatially within each greenhouse compartment and their shoots were placed permanently in transparent cuvettes (Fig. S2a) made from acrylic glass tubes (6.65 L PMMA, Saalberg GmbH, Feldkirchen, Germany). Dismountable acrylic glass caps on the downward-facing side allowed to install the cuvette at the seedlings stem. The inside of the cuvettes had been coated with chemical inert foil FEP (fluorinated ethylene propylene, PTFE Spezialvertrieb, Stuhr, Germany), Small gaps between the stem and the cuvette were sealed with plastic putty (Teroson, Henkel Adhesives, Düsseldorf, Germany) to minimize gas leakage. A fan (412 FM, ebm-papst GmbH & Co. KG, Mulfingen, Germany) guaranteed well-mixing of the air inside the cuvettes. To assess environmental conditions, each cuvette was equipped with a calibrated photo diode (G1118, Hamamatsu Photonics, Hamamatsu, Japan), a calibrated thermocouple (5SC-TTTI-36-2 M, Newport Electronics GmbH, Deckenpfronn, Germany).

The 18 cuvettes (*n* = 4 per treatment, *n* = 2 for empty background) were measured continuously in an automated sequence as follows. After the distal cap of the cuvette had closed, a constant air stream (5 L F-201CZ-10 K, Bronkhorst, Ruurlo, the Netherlands) of clean air with on average 438 ± 3 µmol mol^−1^ [CO_2_] and 6.5 ± 0.1 mmol mol^−1^ [H_2_O] was supplied for 10 min. Zero air was generated by using an oil-free compressor (SLP-07E-S73, Anest Iwata, Yokohama, Japan) connected to an Ultra Zero Air generator (Ultra Zero Air GT, LNI Schmidlin SA, Geneva, Switzerland). CO_2_ was supplied from a gas cylinder and water vapour was added to the air stream via a nebulizing evaporation pump (LCU Liquid Calibration Unit, Ionicon, Innsbruck, Austria). The air supply was channeled through the main tubing made of stainless-steel tubing (3/8 inch Swagelok, Ohio, USA) coated with SilcoNert (Silco Tek GmbH, Bad Homburg, Germany) and gas flow to the cuvettes was controlled by two 2/2-way solenoid valves with a PTFE housing (0121-A-6, 0-FFKM-TE, Bürkert, Ingelfingen, Germany) and PTFE tubing (ScanTube GmbH, Limburg, Germany). The detailed schematic of the measurement system can be found in Bamberger et al. (2017). For background measurements, two empty cuvettes (one each for the control and heat treatment) were measured during each measurement cycle and the recorded data were subtracted from the measurements containing seedlings (Birami et al. 2018).

Differences in [CO_2_] and [H_2_O] between reference air and measurement air leaving the cuvettes were recorded differentially via a LI-7000 connected to a LI-840 (both LI-COR Inc., Lincoln, NE, USA). Net CO_2_ exchange (*A*_net_, *R*_dark_)_,_ transpiration (*E*) and stomatal conductance (*g*_s_) were calculated as previously described in Birami et al. (2018). BVOC fluxes were measured with a high sensitivity proton-transfer-reaction (quadrupole) mass spectrometer (PTR-(Q)MS, IONICON, Innsbruck, Austria). The PTR-MS was operating at standard conditions with a drift tube pressure of 2.3 mbar and a drift tube voltage of 600 V. More detailed settings of the instrument can be found in Bamberger et al. (2017). Volatile compounds were detected on protonated nominal mass ratios (m z^−1^) and quantified using a defined VOC mixture (14 components in nitrogen) of standard gas (#24,182–650 IONICON, Innsbruck, Austria). PTR-MS calibration was performed at ambient humidity with a liquid calibration unit (LCU, IONICON, Innsbruck, Austria) on a weekly basis using a four-step calibration routine at mole fractions of 7.5, 5, 2.5 and 0 ppb.

The average sensitivity and limit of detection for each compound measured are given in Table 2. Since there was no representative for the GLV (e.g. z-3-hexenal) in our standard gas mixture, the sensitivity for C_6_H_10_O on m z^−1^ 99 was estimated to be on average 3.15 ± 0.13 ncps ppb^−1^, derived from the average sensitivity of xylene and toluene (the compound in the standard mixture being closest to m z^−1^ 99 to consider for the transmission efficiency of the quadrupole mass filter) multiplied by 0.33, its known fractionation patterns (33% on m z^−1^ 99) (Fall et al. 1999). This was corrected for reaction rate coefficient k of z-3-hexenal derived from Cappellin et al. (2012) for a E:N ratio of 120 Td (k_toluene_ = 2.08;k_xylene_ = 2.27;k_z-3-hexenal_ = 3.25). The limit of detection for m z^−1^ 99 is hence not given. The sensitivity for C_8_H_8_O_3_ m z^−1^153 (on average 4.96 ± 0.33 ncps ppb^−1^) was derived from a liquid calibration using a calibration mixture of methyl salicylate (A0366376, CAS: 119–36-8, ACROS Organics, New Jersey, USA) in H_2_O (Type 1, MilliQ® Direct8, Merck KGaA, Darmstadt, Germany) (7.8 ppb, 5.2 ppb, 2.6 ppb, 1.3 ppb and 0 ppb). Isoprene could not be distinguished from 2-methyl-3-butene-2-ol (MBO) with our method, hence we did not investigate this compound in detail and address it as “isoprene + MBO”. Ethanol could not be detected in satisfactory quality and was hence not further interpreted (see limit of detection Table 2).

**Table 2:** Sensitivity (± 1SD) and limit of detection of all measured compounds in the calibration standard averaged over all weekly calibration cycles. Sensitivity is defined as normalized counts per second per ppb of primary ions H_3_O^+^ and H_2_OH_3_O^+^ within volume, ncps ppbv^−1^

Compound	Mass fragment	Sensitivity	Limit of detection
		ncps ppbv^−1^	ppbv
Methanol*	33	9.55 ± 0.35	0.35 ± 0.05
Acetaldehyde*	45	14.57 ± 0.63	0.1 ± 0.04
Ethanol	47	0.80 ± 0.11	3.52 ± 0.56
Acetone*	59	15.77 ± 1.12	0.06 ± 0.02
Isoprene (MBO’s)	69	5.10 ± 0.46	0.09 ± 0.03
Monoterpene fragments	81	5.25 ± 0.76	0.07 ± 0.02
Toluene	93	6.66 ± 0.89	0.08 ± 0.02
Hexenal*	99	3.15 ± 0.13	NaN
O-Xylene	107	6.11 ± 0.98	0.11 ± 0.04
Monoterpene (α-pinene)*	137	1.65 ± 0.37	0.13 ± 0.04
Methyl Salicylate*	153	4.96 ± 0.33	0.08 ± 0.02

For calculation of the apparent fluxes, linear interpolation within two calibration cycles was used, hence the table presents a guideline example for the procedure. BVOC analysed are highlighted with an asterisks. BVOCs not present in the calibration standard (#24,182–650 IONICON) are in italic and their respective sensitivities were calculated as described in the methods. Note that m81 was not used in the analyses

To ensure that concentrations of volatiles represented steady-state conditions, emissions were calculated from the last 360 s per 10 min measurement (*c*_out,_ *c*_0_), given that the stability quality criteria were full-filled and backflow from the cuvettes was > 0.3 L min^−1^. Linear regression data were used to assess stability of the signal using an upper boundary for the regression slope ≤ 3 $\left(\frac{\sqrt{\langle s\rangle }}{\delta t}\right)$, where $\langle s\rangle$ is the average signal from the analyzer in cps and $\delta t$ is the length of the time interval used for averaging in s. Note: the term $\sqrt{\langle s\rangle }$ is given by the Poison-Noise of the analyzer. The fluxes of volatile compounds (*E*_ν_ in nmol m^−2^ s^−1^) were calculated from the concentration differences of air leaving the cuvette *c*_out_ and the concentrations leaving an empty chamber *c*_0_ (Niinemets et al. 2011) as follows

1 $$E_{v} = \left( {c_{{{\text{out}}}} - c_{{\text{O}}} } \right)\frac{f}{{l_{{\text{a}}} }}$$

with the flowrate $f$ in mol s^−1^ to each cuvette and the projected leaf area of the seedlings ${l}_{\mathrm{a}}$ in m^2^ (Birami et al. 2018). The projected leaf area was derived from photographs taken during the experiment and estimated through needle color thresholds (Fig. S2b). The projected area was linearly extrapolated.

### Endogenous monoterpene and sesquiterpene measurements

To determine the impacts of two consecutive heatwaves on the pools of endogenous MT and SQT, six additional seedlings per treatment were sampled between 1 and 3 pm on the last day of the second heatwave. The lower part of the seedlings’ stem was removed and the samples (mainly needles and twigs) were immediately frozen in liquid nitrogen and ground in porcelain mortars to a fine powder and stored at  – 80 °C until further processing. MT and SQT were analyzed similarly as done before (Ghirardo et al. 2010; Vanhatalo et al. 2018). One mL of hexane (SupraSolv for GCMS, Merck Chemicals GmbH, Germany) containing 859.3 pmol µL-^1^ of δ-2-carene as the internal standard was added as a solvent to 50 mg of the ground and still frozen plant material in 2 mL gas-tight amber glass vials (Merck) and extracted at 4 °C for 3 h in continuous shaking. Samples were incubated overnight at 5 °C, and the supernatant was transferred into a new 2 mL glass vial using a 1 mL gas-tight syringe (Hamilton). Finally, 1 µL per sample was analyzed by thermo-desorption (TD) gas chromatography mass spectrometry (GC–MS) (Ghirardo et al. 2012). The TD-GC–MS was run and GC–MS data were evaluated as reported elsewhere (Ghirardo et al. 2020). Each sample was analyzed in triplicates and medians were taken from technical replicates. Final endogenous MT or SQT content was related to dried plant material, by weighing for each individual sample, a subsample of the freshly ground and frozen plant powder before and after oven drying at 60 °C for 48 h.

### Data analyses and statistics

To visualize how BVOC emissions vary across treatments, we performed a canonical correspondence analysis (CCA, (TerBraak, Cajo J. F. 1986)) between normalized emission rates, treatment and experimental period. CCA allows to test for the significance of each of the ordination axis, as well as for the influence of factors and cofactors. Monte Carlo permutations (here *n* = 1000) were performed to assess if the variability explained by the model is higher than the variability explained by a randomly generated set of variables (Oksanen 2011). BVOC emission rates were daily-averages that were normalized to pre-stress conditions per individual seedling to reduce the effect of biologic variance. Two separated analyses were performed on the surviving seedlings only (excluding m81, m93, m107, see Table 2): (i) pre-stress vs. first heatwave, and (ii) first heatwave vs. second heatwave. CCA analyses were done using the “Vegan” package, V2.5–6 (Oksanen et al. 2013).

Linear mixed effect models (lme, R packages: “lme4”, “nlme” and “MuMIn”) were used to test for dependencies of BVOC emissions, gas exchange on rSWC or temperature and treatment (fixed effects) Seedling was considered as a random effect. Temporal auto-correlation was accounted for by including a first-order Auto-Regressive model in the lme (Box et al. 2016). AICc criteria (Akaike information criterion corrected for small sample size) was used to select for the most parsimonious model (i.e., best model with as few predictive variables as possible), with a threshold for acceptation of 2, based on (Burnham and Anderson 2004). When the most parsimonious model was identified, a pseudo R^2^ was calculated both for the fixed and the combined fixed and random effects and confidence intervals (CI) given (Nakagawa et al. 2013). Normality of the residuals and homoscedasticity were visually inspected.

During the course of the experiment, three of the continuously measured seedlings (BVOC, gas exchange) in the heat-drought and one in the heat-treatment died due to overheating (Birami et al. 2018). Hence, treatment-specific analyses of BVOC emissions were done using the surviving seedlings only (heat-drought *n* = 1, heat *n* = 3, drought *n* = 4, control *n* = 4). The impact of dying and death on BVOC emissions was analyzed by grouping the heat and heat-drought seedlings into surviving (*n* = 4) and dying (n = 4). In case to overcome tree-specific differences in absolute emission rates, we normalized the data to a tree-specific median derived from before stress-conditions (110–114 DOY), which was for instance done when analyzing mortality responses. Treatment-averaged absolute emission rates per experimental period can be found in the Supplement (Table S4) and are given in Fig. 4.

## Results

### BVOC emissions during heatwaves and drought

**Fig. 2:**
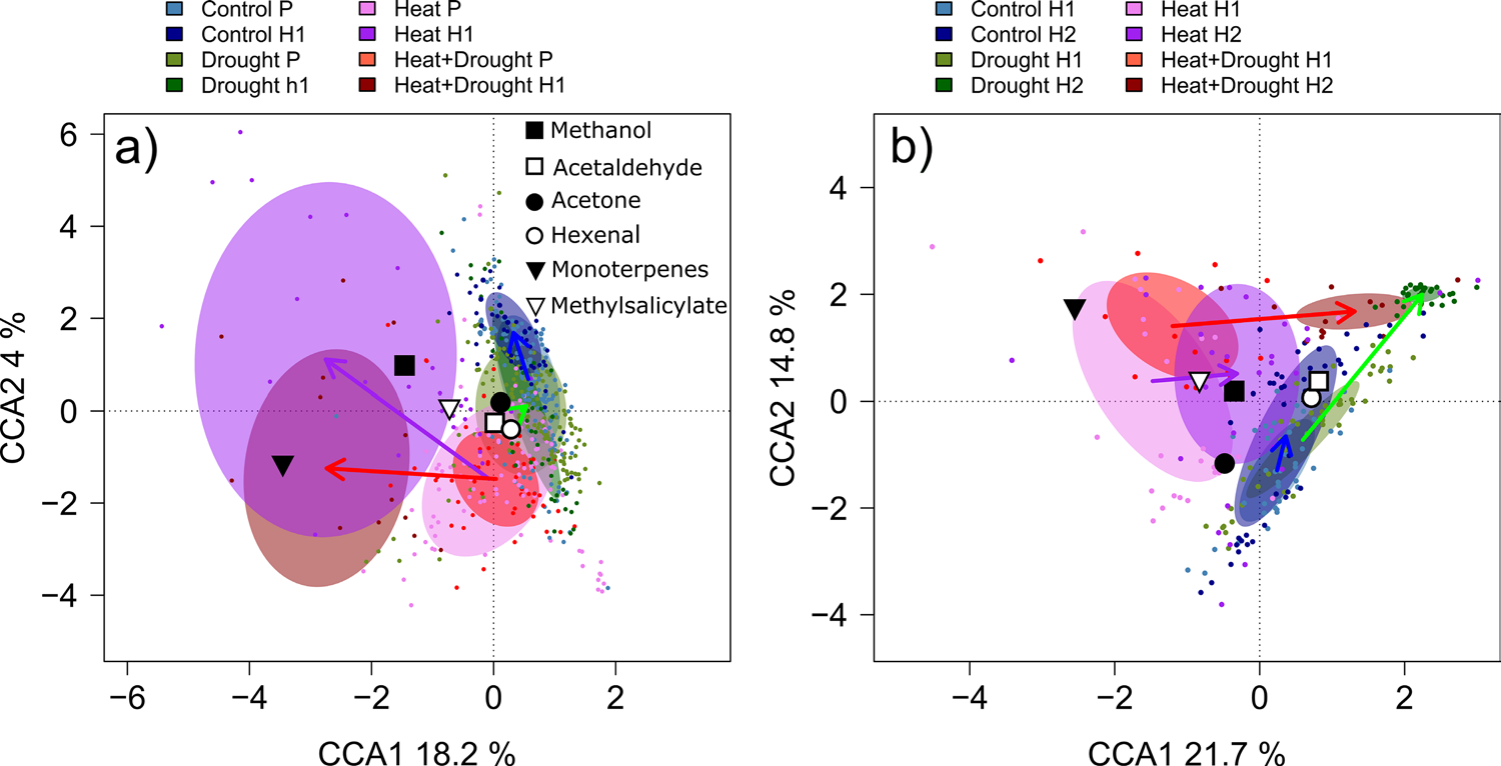
**a** Pre-stress and treatment-specific stress responses of six selected BVOC emissions of the surviving Aleppo pine seedlings (methanol, acetaldehyde, acetone, hexenal, monoterpenes, methyl salicylate). Shown as canonical correspondence analysis (CCA; for details see Data analysis and statistics section) to test for different responses of BVOC emissions of control (blue), drought (green), heat (magenta) and heat-drought (red) seedlings for pre-stress (P, lighter color) and first heatwave (H1, darker, corresponding color) conditions. **b** Comparison of treatment-specific BVOC emission responses between the first (H1) and the second heatwave (H2). Colored areas depict the 95% confidence interval ellipsoid per treatment and period. Dependencies between the compounds (loadings) and the canonical variates (treatment) are shown for methanol, acetaldehyde, acetone, hexenal, monoterpenes and methyl salicylate. Note that emissions are only given for the surviving seedlings (heat: *n*  = 3, heat-drought: *n*  = 1, control: *n*  = 4, drought: *n*  = 4)

Because extreme temperatures were reached during the heatwaves, three of the continuously monitored seedlings in the heat-drought and one in the heat treatment died. Because the dying seedlings experienced different BVOC emissions, we concentrate here on the surviving seedlings first. The emission patterns of the Aleppo pine seedlings in the heat *n* = 3 and heat-drought (*n* = 1) treatment responded similar to heat stress (Fig. 2a). High temperatures predominantly increased emissions of MT and methanol compared to pre-stress conditions. Further, we found the emissions of methyl salicylate and hexenal to increase modestly in response to the heatwave. Interestingly, the drought treatment alone did at first not result in a distinct BVOC response (Fig. 2a), only with the progression of the experiment and increasing soil drought (Fig. 2b).

### Sensitivity of acetone emissions to soil water availability

**Fig. 3:**
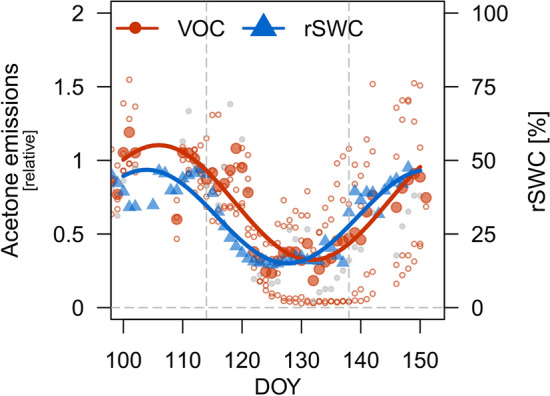
Dynamics of daily-averaged relative soil water content (rSWC [%], blue triangles) and acetone emissions during drying and re-wetting shown for the drought (*n* = 4; open red circles) and heat-drought (*n* = 1; grey solid circles) treatment. Acetone emissions are given relative to the pre-stress for measurements when PAR ≥ 100 (open filled red circles present daily-averages combining both treatments). Colored lines are added to visualize the overall development and represent a trigonometric best-fit using non-linear regression: α*sin(ω*(x + ϕ)) + C with x being DOY and starting parameters (α, ω, ϕ, C)

Here we focus on the impact of drought stress on BVOC emissions and found one distinct response. We found acetone to co-vary with soil water content (Fig. 3) and its emissions to decrease at low water availability (see also Fig. 2b). We found that a 10% decrease in rSWC results in a 10% (5–14% CI; derived from lme) decrease in acetone emissions compared to well-watered conditions. Furthermore, both g_s_ and transpiration were included in the lme model, with acetone emissions being better explained by transpiration (pseudo-R^2^_fixed_ = 0.39) than by g_s_ (pseudo-R^2^_fixed_ = 0.1). Hence, changes in acetone emission were most likely caused by soil water availability and tree water flux. As we observed that acetone emissions from our potting-medium were negligible (< 3% of average acetone emission rates from well-watered seedlings; data not shown), acetone was most likely produced in the root tissues and transported to the shoots via the transpiration stream. No other BVOC showed a clear drought response (Fig. 2; Table S4).

### Impact of repeated heatwaves on gas exchange and BVOC emissions

**Fig. 4:**
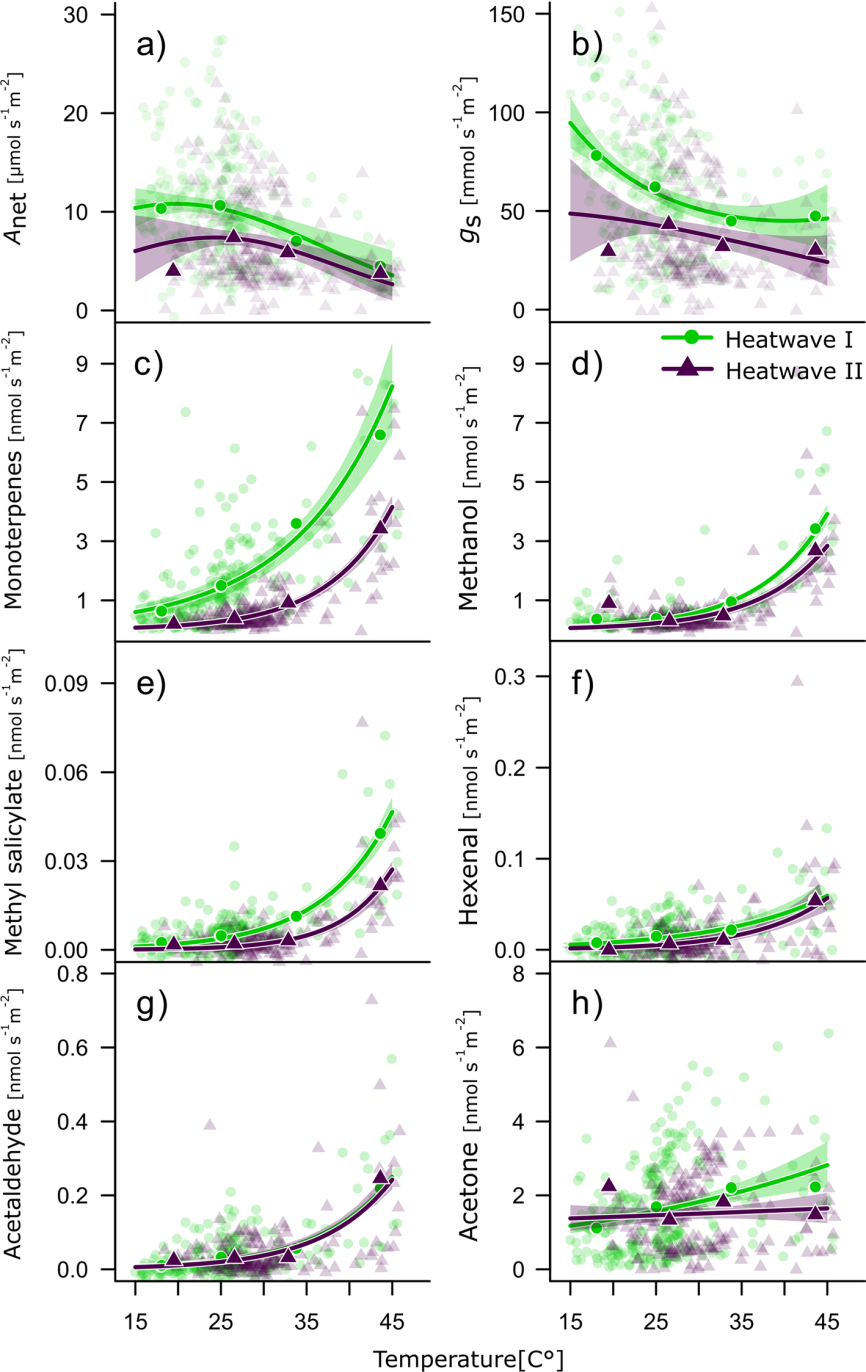
Differences in temperature responses of gas exchange and BVOC emissions during the first (filled circles, light green) and second heatwave (triangles, lilac) of the surviving Aleppo pine seedlings in the heat treatment (*n* = 3). Data are given for PAR ≥ 100 µmol m^−2^ s^−1^ including several days before heatwave initiation. **a** Temperature (T) responses of photosynthesis (A_net_) and **b** stomatal conductance (g_s_,) are shown and the regression lines depict second order exponential functions (exp(a + b(T) + c(T)^2^)). **c** The temperature responses of monoterpenes, **d** methanol, **e** methyl salicylate, **f** hexenal, **g** acetaldehyde and **h** acetone are depicted by exponential functions (exp(b(T) + c). Shaded areas depict the 95% confidence intervals of the fitted functions. Note that VPD increased exponentially with temperature: VPD = exp(0.66(T)-0.85) (R^2^ = 0.94 of log-transformed function). The transparent data points are single measurements, while the solid symbols are bin-averages (10–50 °C by 10 °C) and shown for clarity

We investigated the impact of repeating heatwaves on the temperature response of BVOC emissions and gas exchange in the surviving Aleppo pine seedlings (Fig. 4). *A*_net_ peaked at about 21.5 °C when VPD had reached 1.8 kPa (increase in temperature exponentially increased VPD following (VPD = exp (0.066(T)-0.85), R^2^ = 0.94 of log-transformed function, Fig. S3b), while *g*_s_ decreased to tightly regulate seedling water loss. In contrast, all observed BVOC emissions, except acetone (Fig. 4h), increased with increasing temperatures exponentially (Fig. 4c–g). It is notable that although *g*_s_ decreased in response to the heatwaves, *E* (Fig. S3a) followed an exponential temperature response with slightly lower transpiration rates during the second heatwave.

MT and methanol represented the highest emission rates during heat exposure (Fig. 4c and d). However, an altered temperature relationship became most obvious for MT and MeSa during the second heatwave after exposure to the first heatwave. To test for differences in responses of emissions to temperature, separate lme were computed for MT and MeSa for the first- (H1) and the second heatwave (H2). Although the intercepts of the most parsimonious lme did not differ (overlapping 95% CI, implying similar response amplitudes), the response slope to temperature was reduced markedly (MT, H1: 0.26 [0.21–0.31 CI], H2:0.11 [0.08–0.13 CI]; MeSa, H1:0.001 [0.0009–0.0013 CI], H2:0.0006 [0.0004–0.0008 CI]), which shows that the same temperature does not induce the same emission signal during repeated stress. Methanol, hexenal, and acetaldehyde did not show a change in heat response between heatwaves. Acetone did not show clear temperature dependencies (Fig. 4h).

**Fig. 5:**
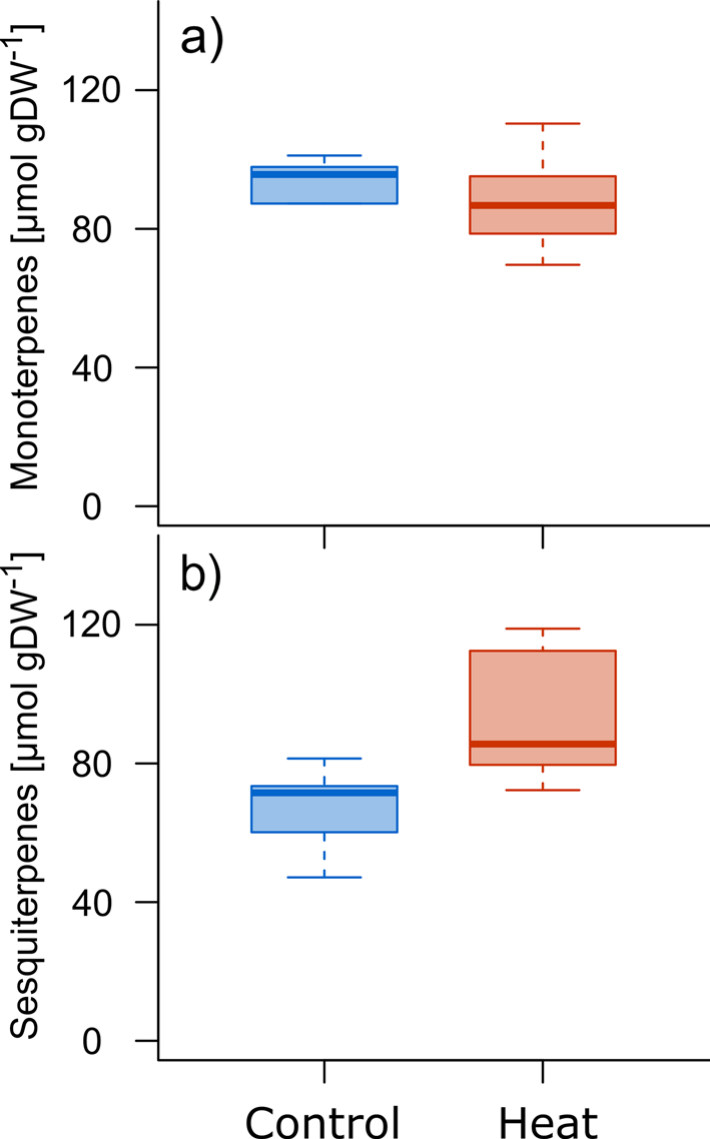
Concentrations of **a** endogenous monoterpenes and **b** sesquiterpenes in green shoots of control and heat-treated Aleppo pine seedlings sampled at the last day of the second heatwave (*n* = 6 per treatment). The box plots depict the median, lower, and upper quartiles (25th–75th percentiles)

We further studied the endogenous MT pools to test the hypothesis that changes in emission patterns might be a consequence of the depletion of MT storages. Total endogenous MT pools did not decrease in plants exposed to two heatwaves, compared to seedlings grown under control conditions (Fig. 5a). The same picture emerged when calculating the total MT pool per average seedling (heat: 148 ± 30SE mg, control: 173 ± 40SE mg based on the molar weight of a-pinene of 136.2 g mol^−1^). This was in contrast to the cumulative MT emissions during the two heatwaves (DOY 114–131) which summed up to 28.1 ± 4.4SE mg (Table S2), and would hence account for a 19.7 ± 1.2SE % decrease of the total pool (as derived above). Considering specific compounds, we did not observe a significant impact of heat stress on any of the 14 MT measured (Table S2). Notably, the overall content of stress-induced SQT compounds (sum of 14) tended to increase compared to the control seedlings (*P* = 0.07, TukeyHSD, Fig. 5b).

### Impacts of tree mortality on BVOC emissions

**Fig. 6:**
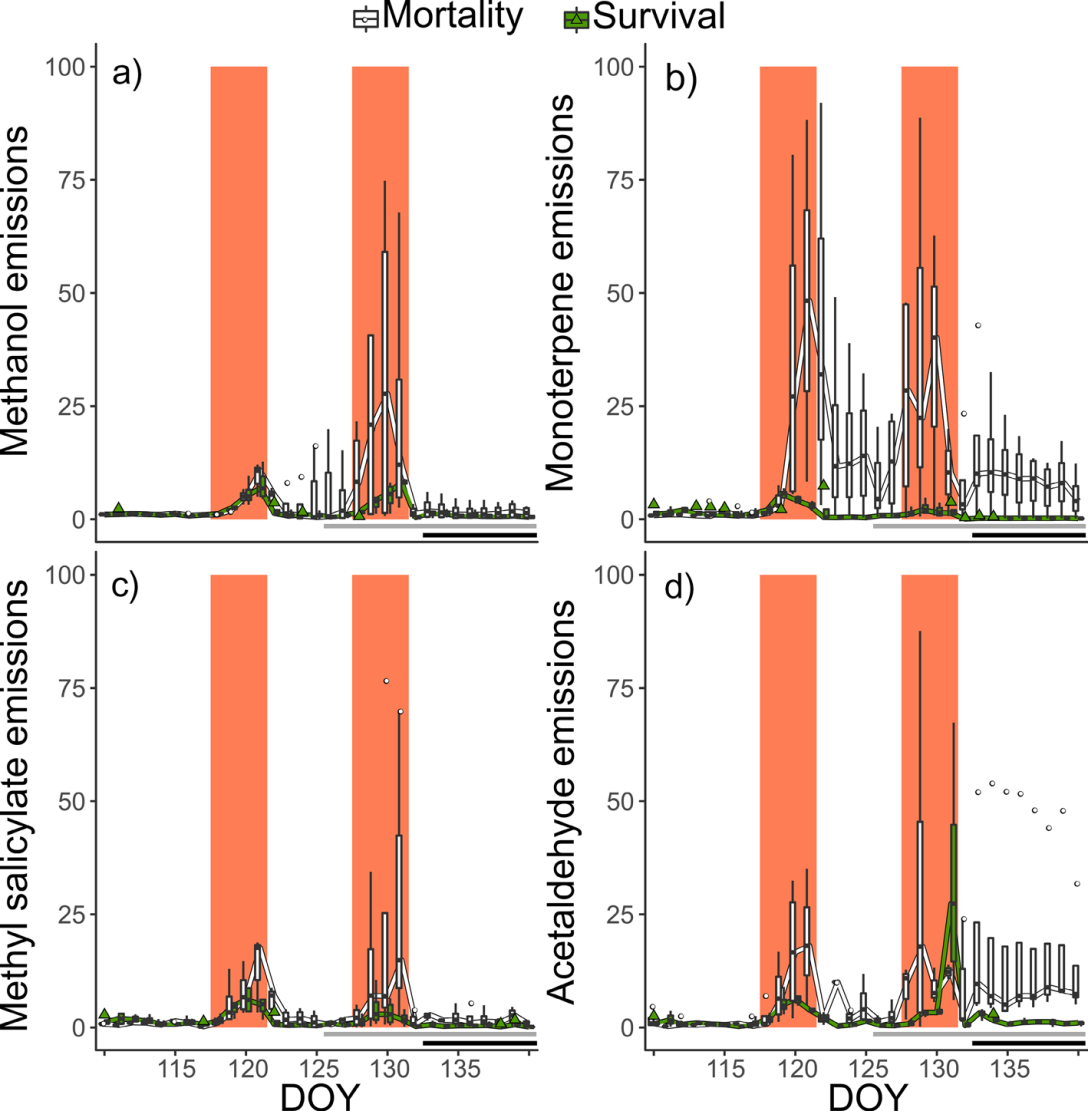
Responses of **a** methanol, **b** monoterpene, **c** methyl salicylate and **d** acetaldehyde emissions during seedling mortality. The emission data are shown relative to pre-stress derived from daily-averages (for measurements when PAR ≥ 100) per seedling, separated in surviving (green, *n* = 4; including 3 seedlings from the heat and 1 seedlings from the heat-drought treatment) and dying (white, *n* = 4; including 1 seedlings from the heat and 3 seedlings from the heat-drought treatment). The two heatwaves are highlighted by a solid colored background (DOY 118–121; 128–131). Horizontal grey bars mark the time course on when either daily-averaged transpiration or net photosynthesis or both of the dying seedlings reached zero. The time when dark respiration reached zero and seedlings were clearly dead is indicated by the black horizontal bars. Daily-averaged emission data was derived for measurements of PAR ≥ 100

The stress intensity, in particular in the combination of heat stress with drought, resulted in pronounced seedling mortality due to overheating. This overheating was more pronounced in the heat-drought treatment due to the lack of evaporative cooling and needle temperatures of 47 °C were reached (Birami et al. 2018). As a consequence some of the seedlings that were constantly monitored for BVOC emissions died: one in the heat treatment and three in the heat-drought treatment. The first indication for a reduced vitality of the seedlings can be seen in net photosynthesis (*A*_net_) and transpiration to reach zero between the two heatwaves (Fig. 6; horizontal grey bars) (Birami et al. 2018). However, we found clear differences in the emission rates between surviving and dying seedlings already much earlier. We found particularly strong responses in acetaldehyde (Fig. 6d, TukeyHSD: P ≤ 0.05) and MT (Fig. 6b, TukeyHSD: P ≤ 0.05) emissions, which were much higher during the first heatwave in the later dying compared to the surviving seedlings. Interestingly, these emissions also remained elevated during the first recovery period (between heatwaves). For methanol (Fig. 6a) and MeSa (Fig. 6c) emissions we found similar responses, albeit with a shift to increased emissions of the dying seedlings during the second heatwave. A clear indication for the death of the seedlings was the moment when dark respiration ceased (Fig. 6, black bar represents when shoot respiration reached zero). This was directly after the end of the second heatwave. Further, analysis of shoot water content at the end of the experiment confirmed the differentiation in mortality and surviving seedlings. Surviving seedlings had an absolute shoot water content of 64.2 ± 2%, while the water content in the dead seedlings averaged at 24.2 ± 13% (Birami et al. 2018).

## Discussion

### Impacts of two consecutive heatwaves on BVOC emissions and gas exchange

We found a strong stimulation of BVOC emissions during two consecutive heatwaves. Monoterpene, methanol, MeSa, hexenal, and acetaldehyde emissions increased exponentially with increasing temperatures. In particular, MT emissions showed a sharp increase between 30 and 43 °C. In contrast, acetone was insensitive to changes in temperature but responded to soil water availability (see below).

Monoterpene emissions are known to respond strongly to temperature increase by release from specific storage pools depending on diffusion parameters and volatility (Kleist et al. 2012; Peñuelas and Llusià 1999; Staudt et al. 2017), including leaves and stems (Vanhatalo et al. 2020). On the other hand, MT emissions of pines have been shown to originate to more than 50% directly from photosynthetic products under stressed conditions (Ghirardo et al. 2010; Taipale et al. 2011). In our experiment, the response of MT emissions of surviving seedlings to temperature was considerably weaker during the second heatwave (emission rates at a specific temperature were about 50%, and maximum emission tended to be lower than during the first heatwave). With this respect, the results from our study agree well with previous studies on MT and MeSa emissions in Scots pine (Kleist et al. 2012), which reported a down-regulation of emissions during consecutive heatwaves. Such a response may indicate depletion of storages or could point towards metabolic adjustments of volatile biosynthesis.

The MT storage in plants, formed by specialized organs such as resin ducts, is typically large. In our investigations, we have found the monoterpene content to be 1.3% of dry weight in shoot biomass of Aleppo pine seedlings (Table S5), similar to those of dried needle biomass of Scots pine (0.8%) reported by Vanhatalo et al. (2018). Usually, a depletion of such a large storage is unlikely to happen in short time (few days), as it did not occur in two years old needles after MT emissions of an entire season (Vanhatalo et al. 2018). However, the depletion of MT storages has been proposed as a possible mechanism if plant tissue is exposed to high temperatures over a longer period (days to months) (Schurgers et al. 2009) and are shown to be reduced after 4 years of heat treatment (+ 3.5 °C) compared to non-treated Douglas fir saplings (Snow et al. 2003). Our measurements showed that the overall MT pools remained unaffected, although the cumulative MT emissions during both heatwaves were expected to deplete approximately 20% of the total endogenous MT pool of the heat-treated seedlings (Table S5). This implies that MT’s are de novo produced and directly released during heatwaves. Possibly, de novo MT production was impaired along with photosynthesis during the first heatwave and this caused lower MT emission during the second heatwave, compared to the first one. Although older investigations could not find a significant contribution of emission from de novo biosynthesis for Aleppo pine (Peñuelas and Llusià 1999), more recent studies indicate that the contribution could be around 50% (Llusia et al. 2016) and might be as high as 70% in 3–4-year-old trees (Staudt et al. 2017). It has also been shown that MT biosynthesis, in general, is sensitive to stress which also negatively affects *A*_net_—such as drought—(Kleist et al. 2012; Lüpke et al. 2017) since the plastidic intermediate for the terpenoid synthesis is closely connected to photosynthesis (Ghirardo et al. 2014). Another explanation for the observed burst of MT during the heatwaves may be that those emissions originated from MT pools of non-specialized structures such as the lipid phase of cellular compartments and membranes (Joensuu et al. 2016; Nagegowda 2010; Yazaki et al. 2017). Overall their amount is small compared to the pool in specific storage tissue such as resin ducts (Ormeño et al. 2011), but might face less diffusive resistance and can readily enter the gas phase. Following this, intramembrane or cuticular MT are likely to be primarily released during the first heatwave and were not refilled before the second heatwave, contributing to the overall smaller emission rates. Hence we have no information about the actual compartmentation of the endogenous MT, de novo synthesized MT may have prevented total pool depletion and a delayed refilling of the non-specialized pools may have reduced the emissions during the second heatwave. As reported in poplar, membrane collapse caused by severe heat stress may as well cause such a burst of several BVOC, including MT (Behnke et al. 2013).

Methyl salicylate emissions, which are known to increase under stress in many tree species (Filella et al. 2006; Joó et al. 2011), also showed an altered temperature-response during the second heatwave. While MeSa has been shown to be released under biotic stress, it has also been related to drought (Karl et al. 2008; Scott et al. 2019). As a signal-transmitting metabolite, MeSa is freshly mobilized from precursor molecules (Heil and Ton 2008). The reduced temperature-response of emissions during the second heatwave might thus indicate a suppression of enzymatic activity and might support the hypothesis that, at least, parts of the observed MT burst are caused by de novo production. Suppression of MeSa mobilization from salicylic acid at high temperatures has been previously reported (Shulaev et al. 1997), while in a study on *Arabidopsis thaliana* thermal pre-treatment (38 °C) induced salicylic acid accumulation (non-volatile form of MeSa) that caused thermotolerance of the plants at 47 °C (Clarke et al. 2004).

Overall, we could show that consecutive heatwaves alter the temperature-sensitivity of MT and MeSa emissions and that these changes are not related to any declines of pools in specific structures. Instead, the decrease in responsiveness seems strongly related to reduced metabolic activity, possibly due to reduced enzyme production or increased damage of metabolic production chains.

### Impact of drought on BVOC emissions

Previously observed responses of BVOC emissions to water availability are inconclusive in the case of Aleppo pine*.* Some studies report MT emissions to decrease with drought (Blanch et al. 2007; Llusia et al. 2016), while others report increases (Llusià et al. 2008). Overall, the emission response might particularly depend on the severity and duration of drought, increasing at first under mild drought but decreasing with drought progression (Ormeño et al. 2007). In our experiments, we could not observe drought-specific responses of MT emissions, which, however, might be due to a physiological adjustment that reduced MT emissions already under non-stressed conditions. The same response has been shown for mature trees at the same site, which is different to stands under less extreme conditions (Llusia et al. 2016), indicating that the adjustment origins from genetic rather than morphological changes.

On the other hand, we found that reduced acetone emissions were indicative for drought conditions. Acetone represented one of the most abundant volatile compounds emitted. Acetone probably originated from the root tissue and was then transported to the shoots of the seedlings as long as soil water availability is sufficient. This is similar to what has been reported for Aleppo pine stands (Filella et al. 2009) and is also supported by studies demonstrating that the emissions of water soluble short-chained compounds depends on *E* (Rissanen et al. 2018). Acetone is the smallest ketone of the anoxic fermentation chain derived from Acetyl-CoA that is metabolized either from pyruvate or fatty-acid oxidation (Fall 2003; Grote et al. 2019) and is indeed easily water-soluble. It was found to be an indicator for flooding in the roots of trees from the Amazonian floodplains (Bracho-Nunez et al. 2012). In temperate and boreal forests, acetone emissions have been generally related to water availability (Janson and Serves 2001; Shao and Wildt 2002). Acetone is thus a likely candidate for an indicator of drought-stress, particularly for coniferous forests. This largely supports our first hypothesis that responses of specific BVOC emissions can be directly linked to the type of stress. Where MT emissions were found to be strongly induced by heat stress, acetone emissions were found to decline under drought.

### Methanol as an indicator for lethal stress

At the end of the experiment, four of the seedlings constantly monitored for gas exchange and BVOC emission died (heat: *n* = 1; heat-drought: *n* = 3). Mortality was more pronounced in the group of heat-drought-stressed seedlings, possibly due to damages from higher leaf temperatures caused by reduced evaporative cooling (> 47 °C, (Birami et al. 2018)). While the exact time of death is challenging to determine, gas exchange data show that *A*_net_ and *E* reached zero between the two heatwaves (Table S3) and that shortly afterwards also dark respiration stopped, indicating that shoots became metabolically inactive. We found an increase of methanol emission in dying (but not in surviving) seedlings one day before this occurrence, while otherwise the emission rate was relatively stable as highlighted before (Seco et al. 2015). To the best of our knowledge, it is the first time that changes in emission patterns of BVOCs were directly linked to heat-induced mortality.

It has been commonly observed that emission of green leave volatiles and oxygenated VOCs such as methanol increase during heat stress (Kleist et al. 2012). This has been particularly reported under high temperatures and limited water supply (Filella et al. 2009; Jardine et al. 2015). Methanol is produced in considerable amounts during cell wall formation, released by pectin methylesterases (PMEs), allowing to adjust the rigidity of the cell walls. Heat stress (35–65 °C) in turn, was reported to activate apoplastic PMEs (Wu et al. 2018). While methanol is being cleaved from pectine, Ca^2+^ is being released, which passes the cell membrane and starts a cascade of intracellular stress signals (Dorokhov et al. 2018; Wu et al. 2018). Thus, excess in methanol production can be a sign of active growth processes, or be the consequence of the demethylation of several methylated compounds (e.g. DNA, RNA, histones, and other proteins) occurring after cell damage and oxidative stress as it was reviewed by Kim et al. (2015). The co-occurrence of a substantial increase in the emissions of lipoxygenase products such as hexenal (Fig S3) with high methanol fluxes (Fig. 6a), provides a strong indication that methanol emissions were related to cell and membrane damages. Indeed, after membrane disruption, lipoxygenase products are formed when the fatty acid substrates trapped in the cell membrane get in contact with cytosolic enzymes. In turn, the concomitant but stronger methanol emissions might be a reliable indicator of lethal heat dosage (Turan et al. 2019).

### Excessive MT and acetaldehyde emissions anticipate higher sensitivity to stress

Monoterpene and acetaldehyde emissions of seedlings that died after the first heatwave were distinct from surviving seedlings, albeit photosynthetic rates were not showing apparent differences between dying and surviving individuals until the first day of stress release (Table S3, S4 (Birami et al. 2018)). Susceptible seedlings showed a tendency of higher emissions in MeSa, 69 m z-1 (which is an unknown compound that could either be isoprene or MBO, Fig. S3a), and hexenal (Fig. S3b).

MeSa and hexenal have been previously depicted as stress sensing and stress signaling molecules (Tawfik et al. 2017; Wu et al. 2018). Acetaldehyde emissions, however, were found to be closely related to stomatal conductance in Aleppo pine (Filella et al. 2009; Seco et al. 2008). This is in contrast to our results, where increasing temperatures decreased stomatal conductance both in surviving and dying seedlings, while acetaldehyde emission rates increased independent of *g*_s_. Increased acetaldehyde production may result from a possible pyruvate substrate overflow mechanism during times of sudden changes in light intensity, when downstream processes of carbohydrate reduction are limited (Karl et al. 2002). Carbohydrate metabolism can also be limited by temperature stress, which is supported by similarities between the metabolic response to anoxia and high temperatures, both inducing anoxic fermentation pathways (Pucciariello et al. 2012) that eventually result in acetaldehyde formation (Kreuzwieser et al. 1999). Formation and production not necessarily result in immediate emission, nonetheless, acetaldehyde is released when terminal cell damage occurs (Behnke et al. 2013; Fall et al. 1999; Loreto et al. 2006; Portillo-Estrada et al. 2015; Turan et al. 2019). Interestingly, also the surviving seedlings showed a higher emission rate of acetaldehyde at the end of the second heatwave, indicating (i) that some damages occurred also in the surviving seedlings which might have been fatal in a third heatwave, and (ii) that acetaldehyde might be a better sensitivity indicator than MT alone.

Still, it is difficult to explain why the dying seedlings had higher acetaldehyde and MT emissions before and after actual mortality happened (Fig. 6b and d) although similar observations have been made on different plants or tissues. For example, high emissions of acetaldehyde and other oxygenated VOCs have been reported during the process of grass drying (Gouw et al. 1999). Also, active dehydrogenases that might produce acetaldehyde have been found in dry heartwood (Tohmura et al. 2012) and during industrial drying of pinewood (McDonald et al. 2002). In all cases, it was assumed that acetaldehyde emissions were caused by enzymatic or microbial remnant activity or oxidative decomposition of cellulose i.e., resin components. Regarding the increased MT emissions, it should be considered that the absolute magnitude of emissions from the dying seedlings (55.2 ± 21.7 mg) doubled the cumulative emissions from surviving heat-treated seedlings (28.1 ± 4.4 mg). This would relate to an approximate loss of about 35% in relation to the stored compounds (Table S5), if assume the pool size of an average seedling. Therefore, and because no seedlings died during the same period, it is unlikely that resin ducts have been significantly damaged and were the origin of the MT burst. However, decreased membrane integrity of more sensitive individuals may have facilitated the release of non-specific MT. Furthermore, MT precursors can be formed in the plastidic methylerythritol phosphate pathway (MEP) (Zeidler and Lichtenthaler 2001). As the MEP pathway is promoted under heat and light stress (Harley et al. 1998), and is well coupled to the abscisic acid sensing network (Asad et al. 2019), increased synthesis of MT by dying seedlings could be explained by a higher stress sensitivity. In summary, we found that the onset of lethal stress induces a distinct BVOC emission composition, which was already detectable about 14 days before mortality actually occurred. This indication of mortality started considerably earlier than any clear trend in gas exchange rates.

Concluding, we found VOC emissions from Aleppo pine seedlings to respond specifically to stress-type and stress-frequency. The reduced temperature sensitivity of monoterpene emissions during a second heatwave was not related to storage depletion in shoot tissues, but likely caused by stress-induced impairment of de novo synthesis and intra-tissue localization. Moreover, we found a distinct response in MT, acetaldehyde emissions to precede seedling mortality with methanol as an indicator for lethality under heat and combined heat-drought stress. We did, however, not investigate different ontogenetic stages, which means that a species might acclimate to heatwaves and drought conditions and that emission responses of adult individuals might differ from those of seedlings. In fact, eddy-covariance measurements in different Aleppo pine stands indicate that emissions at the Yatir forest are smaller than at stands with less extreme conditions (Seco et al. 2017), which could be interpreted as a long-term acclimation. Understanding these signals, might help optimizing management for stress mitigation in precision farming or tree planting (Kravitz et al. 2016; Lüttge and Buckeridge 2020), and pave the way to new conceptual modelling frameworks towards characterizing stress severity related to tree mortality.

## Supplementary Information

Below is the link to the electronic supplementary material.

Supplementary file1 (DOCX 3903 KB)

## Data Availability

The dataset generated during and/or analysed during this study is made available in the Repository PANGAEA, https://doi.pangaea.de/10.1594/PANGAEA.923768*.*
