# Supplementary material for: Heatwave frequency and seedling death alter stress-specific emissions of volatile organic compounds in Aleppo pine
Source: Oecologia. 2021 Apr 9;197(4):939–56. doi: 10.1007/s00442-021-04905-y (PMC8591014; doi:10.1007/s00442-021-04905-y)
Supplement: Supplementary file 1 — Supplementary file1 (DOCX 3903 KB) [file 442_2021_4905_MOESM1_ESM.docx]

**Heatwave frequency and seedling death alter stress-specific emissions of volatile organic compounds in Aleppo pine.**

Benjamin Birami (1,4), Ines Bamberger (2), Andrea Ghirardo (3), Rüdiger Grote (1), Almut Arneth (1), Elizabeth Gaona-Colmán (1), Daniel Nadal-Sala (1), and Nadine K. Ruehr (1)

(1) Karlsruhe Institute of Technology KIT, Institute of Meteorology and Climate Research – Atmospheric Environmental Research, 82467 Garmisch-Partenkirchen, Germany (benjamin.birami@kit.edu)

(2) University of Bayreuth, Bayreuth Center of Ecology and Environmental Research (BayCEER), Atmospheric Chemistry, Dr.-Hans-Frisch-Straße 1-3, 95448 Bayreuth, Germany

(3) Research Unit Environmental Simulation, Institute of Biochemical Plant Pathology, Helmholtz Zentrum München, Ingolstädter Landstr. 1, 85764, Neuherberg, Germany.

(4) University of Bayreuth, Chair of Plant Ecology, Universitätsstraße 30, 95440 Bayreuth, Germany (benjamin.birami@uni-bayreuth.de)

Supplemental Material:

Table S1 Shown are daily intervals of environmental control parameters of the glasshouse facility. Values depict Monthly averages ±1SE of half hourly data during the cultivation of the P. halepensis seedlings.

| Interval | | | Temperature | | | Relative humidity | | | | Photos. active radiation | | |
| --- | --- | --- | --- | --- | --- | --- | --- | --- | --- | --- | --- | --- |
| Hour of day | Month | Year | [°C] | | | [%] | | | | µmol m^-2^s^-1^ | | |
| 0-6 | 7 | 2015 | 17.8 | ± | 1.3 | | 74.8 | ± | 3.6 | 46.6 | ± | 122.4 |
| 6-9 | 7 | 2015 | 22.5 | ± | 1.3 | | 60.3 | ± | 5.2 | 710.1 | ± | 359.3 |
| 9-12 | 7 | 2015 | 24.4 | ± | 0.9 | | 50.9 | ± | 2.3 | 1044.8 | ± | 353.1 |
| 12-15 | 7 | 2015 | 23.9 | ± | 1.2 | | 54.5 | ± | 6.4 | 834 | ± | 478.2 |
| 15-18 | 7 | 2015 | 21.9 | ± | 1.2 | | 64.6 | ± | 6.4 | 385.3 | ± | 324.9 |
| 18-21 | 7 | 2015 | 19.5 | ± | 1.4 | | 73.1 | ± | 5.1 | 8.1 | ± | 57.6 |
| 21-0 | 7 | 2015 | 17.9 | ± | 1.4 | | 75.3 | ± | 4.1 | 0 | ± | 0.6 |
| 0-6 | 8 | 2015 | 16.3 | ± | 2.1 | | 74.7 | ± | 3.9 | 14.3 | ± | 77.8 |
| 6-9 | 8 | 2015 | 22 | ± | 2.6 | | 60.9 | ± | 9.7 | 658.3 | ± | 586.9 |
| 9-12 | 8 | 2015 | 25.1 | ± | 1.4 | | 48.3 | ± | 7.1 | 975.8 | ± | 319.7 |
| 12-15 | 8 | 2015 | 25.7 | ± | 1.9 | | 48.9 | ± | 6.4 | 925.8 | ± | 611.2 |
| 15-18 | 8 | 2015 | 23 | ± | 1.8 | | 59.2 | ± | 5.9 | 299 | ± | 208.4 |
| 18-21 | 8 | 2015 | 19.5 | ± | 1.8 | | 72 | ± | 6 | 0 | ± | 19 |
| 21-0 | 8 | 2015 | 17.4 | ± | 1.8 | | 74.1 | ± | 4.7 | 0 | ± | 0.9 |
| 0-6 | 9 | 2015 | 14.8 | ± | 2.6 | | 68.1 | ± | 5.3 | 0 | ± | 37.6 |
| 6-9 | 9 | 2015 | 19.9 | ± | 3.4 | | 58.5 | ± | 9.9 | 621.6 | ± | 546.6 |
| 9-12 | 9 | 2015 | 24.6 | ± | 3.1 | | 45.5 | ± | 8.2 | 935 | ± | 651.5 |
| 12-15 | 9 | 2015 | 24.8 | ± | 2.7 | | 44.3 | ± | 5.6 | 910.7 | ± | 603.8 |
| 15-18 | 9 | 2015 | 20.5 | ± | 2.7 | | 57 | ± | 7.3 | 184.5 | ± | 255.2 |
| 18-21 | 9 | 2015 | 16.2 | ± | 1.3 | | 68.2 | ± | 5.3 | 0 | ± | 0.9 |
| 21-0 | 9 | 2015 | 15 | ± | 1.7 | | 68.6 | ± | 4.4 | 0 | ± | 1.1 |
| 0-6 | 10 | 2015 | 16.6 | ± | 0.5 | | 61.9 | ± | 4.6 | 0 | ± | 11.6 |
| 6-9 | 10 | 2015 | 19.8 | ± | 2.2 | | 55.9 | ± | 7.6 | 316.9 | ± | 238.4 |
| 9-12 | 10 | 2015 | 22.9 | ± | 2.6 | | 47.2 | ± | 7.3 | 660.5 | ± | 245.6 |
| 12-15 | 10 | 2015 | 21.7 | ± | 2 | | 52.4 | ± | 5 | 728.1 | ± | 576.8 |
| 15-18 | 10 | 2015 | 18.6 | ± | 1.2 | | 62.7 | ± | 4.2 | 55.9 | ± | 114.9 |
| 18-21 | 10 | 2015 | 16.3 | ± | 0.3 | | 64.1 | ± | 4.7 | 0 | ± | 0.1 |
| 21-0 | 10 | 2015 | 15.8 | ± | 0.1 | | 63.9 | ± | 4.9 | 0 | ± | 0.3 |
| 0-6 | 11 | 2015 | 13.7 | ± | 1.2 | | 58 | ± | 4.5 | 0 | ± | 2.1 |
| 6-9 | 11 | 2015 | 16.3 | ± | 1.8 | | 49.9 | ± | 8.9 | 140 | ± | 225 |
| 9-12 | 11 | 2015 | 19.8 | ± | 2.6 | | 38.8 | ± | 8.1 | 501.7 | ± | 250.2 |
| 12-15 | 11 | 2015 | 20.4 | ± | 2.3 | | 39.3 | ± | 10.3 | 331.1 | ± | 231.3 |
| 15-18 | 11 | 2015 | 18.4 | ± | 1.5 | | 49.2 | ± | 10.8 | 0 | ± | 27.2 |
| 18-21 | 11 | 2015 | 15.2 | ± | 1.4 | | 57.1 | ± | 6.4 | 0 | ± | 0 |
| 21-0 | 11 | 2015 | 13.9 | ± | 1.3 | | 57.9 | ± | 4.9 | 0 | ± | 0.4 |
| 0-6 | 12 | 2015 | 12.5 | ± | 0.6 | | 50.7 | ± | 3 | 0 | ± | 2.5 |
| 6-9 | 12 | 2015 | 15.8 | ± | 1.2 | | 39.5 | ± | 4.3 | 56.2 | ± | 63.9 |
| 9-12 | 12 | 2015 | 18.2 | ± | 1.9 | | 34.9 | ± | 8.6 | 292.3 | ± | 150.8 |
| 12-15 | 12 | 2015 | 19.6 | ± | 1.4 | | 33.5 | ± | 7.8 | 190.7 | ± | 111.8 |
| 15-18 | 12 | 2015 | 18.4 | ± | 1 | | 38.4 | ± | 4.7 | 0 | ± | 15.7 |
| 18-21 | 12 | 2015 | 13.1 | ± | 0.8 | | 46.9 | ± | 6.3 | 0 | ± | 0 |
| 21-0 | 12 | 2015 | 12 | ± | 0.2 | | 47.8 | ± | 6.3 | 0 | ± | 0.2 |
| 0-6 | 1 | 2016 | 9.9 | ± | 1.8 | | 54 | ± | 4.7 | 0 | ± | 2.1 |
| 6-9 | 1 | 2016 | 13.1 | ± | 1.9 | | 48 | ± | 6.8 | 76 | ± | 111.3 |
| 9-12 | 1 | 2016 | 17.3 | ± | 3.1 | | 43.6 | ± | 10.7 | 443.2 | ± | 274.3 |
| 12-15 | 1 | 2016 | 18.1 | ± | 3.8 | | 44.2 | ± | 11.9 | 332.6 | ± | 246.1 |
| 15-18 | 1 | 2016 | 15 | ± | 2.4 | | 50 | ± | 7.7 | 2.6 | ± | 40.5 |
| 18-21 | 1 | 2016 | 11.4 | ± | 1.3 | | 53.1 | ± | 6.9 | 0 | ± | 1.1 |
| 21-0 | 1 | 2016 | 10.2 | ± | 1.4 | | 53 | ± | 6.3 | 0 | ± | 1.1 |
| 0-6 | 2 | 2016 | 8.9 | ± | 1.5 | | 55.2 | ± | 5.1 | 0 | ± | 1.6 |
| 6-9 | 2 | 2016 | 13.4 | ± | 2.8 | | 50.8 | ± | 5.6 | 158.5 | ± | 160.1 |
| 9-12 | 2 | 2016 | 19 | ± | 3.7 | | 40.9 | ± | 11.1 | 585.2 | ± | 240.5 |
| 12-15 | 2 | 2016 | 19.8 | ± | 4.9 | | 37.1 | ± | 12.4 | 414.7 | ± | 170.7 |
| 15-18 | 2 | 2016 | 14.9 | ± | 2.6 | | 47.9 | ± | 8.1 | 52.2 | ± | 104 |
| 18-21 | 2 | 2016 | 10.6 | ± | 1.7 | | 52.2 | ± | 5.8 | 0 | ± | 0.8 |
| 21-0 | 2 | 2016 | 9.1 | ± | 1.3 | | 53.9 | ± | 5.1 | 0 | ± | 0.8 |

Table S2: List of all TD-GC-MS quantified endogenous terpenoids from biomass samples of only surviving seedlings at the end of the second heatwave (*n*=6 per treatment, Control, Heat). Monoterpenes and Sesquiterpenes were summarized in MT_sum and SQT_sum. Values are averages ± 1SE. These data were used to generate Fig 5 in the main document.

| Component | Control | | | Heat | | |
| --- | --- | --- | --- | --- | --- | --- |
| Monoterpene [µmol gDW^-1^] | | | | | | |
| Tricyclene | 0.87 | ± | 0.04 | 0.84 | ± | 0.07 |
| a-Pinen | 47.26 | ± | 2.50 | 44.14 | ± | 3.12 |
| Camphene | 0.33 | ± | 0.01 | 0.31 | ± | 0.02 |
| Sabinen | 0.94 | ± | 0.21 | 0.76 | ± | 0.15 |
| b-Pinen | 2.42 | ± | 0.54 | 3.23 | ± | 0.87 |
| Myrcen | 2.39 | ± | 0.70 | 1.98 | ± | 0.70 |
| d-3-carene | 26.80 | ± | 5.80 | 28.62 | ± | 4.50 |
| Limonen | 1.88 | ± | 0.21 | 2.10 | ± | 0.09 |
| b-Phellandren | 1.65 | ± | 0.05 | 1.71 | ± | 0.12 |
| g-Terpinene | 0.34 | ± | 0.07 | 0.33 | ± | 0.06 |
| a-Terpinolene | 3.19 | ± | 0.70 | 2.94 | ± | 0.53 |
| allo-Ocimene | 0.07 | ± | 0.01 | 0.03 | ± | 0.01 |
| Bornylacetate | 0.98 | ± | 0.19 | 0.49 | ± | 0.26 |
| 03_Geranylacetate | 0.25 | ± | 0.05 | 0.22 | ± | 0.10 |
| Sesquiterpene [µmol gDW^-1^] | | | | | | |
| 02_SQT | 0.01 | ± | 0.00 | 0.02 | ± | 0.01 |
| 01_d-Elemene | 0.19 | ± | 0.04 | 0.11 | ± | 0.04 |
| 02_b-Elemene | 0.76 | ± | 0.20 | 1.23 | ± | 0.16 |
| 03_b-Cubebene | 0.88 | ± | 0.19 | 1.06 | ± | 3.15 |
| Caryophyllene+SQT | 40.72 | ± | 5.46 | 60.77 | ± | 4.56 |
| 05+06_b-Cubebene+ trans-b-Farnesene_S | 1.03 | ± | 0.19 | 0.85 | ± | 0.12 |
| 03_SQT | 0.16 | ± | 0.06 | 0.59 | ± | 0.13 |
| 07_e-Muurolene | 0.33 | ± | 0.07 | 0.26 | ± | 0.05 |
| a-Humulene | 7.10 | ± | 0.96 | 10.96 | ± | 0.87 |
| 04_SQT | 1.14 | ± | 0.26 | 0.77 | ± | 0.18 |
| 09_a-Muurolene | 7.58 | ± | 1.08 | 7.72 | ± | 0.83 |
| 05_SQT | 0.36 | ± | 0.06 | 0.24 | ± | 0.05 |
| 11_cis-alpha-Bisabolene | 0.18 | ± | 0.02 | 0.33 | ± | 0.03 |
| 01_Elemol | 3.09 | ± | 0.74 | 2.78 | ± | 0.42 |
| MT_sum | 95.68 | ± | 6.68 | 86.76 | ± | 5.88 |
| SQT_sum | 71.52 | ± | 5.96 | 85.59 | ± | 7.67 |

Table S3: Shown are Transpiration *E*, net photosynthesis *A*_net_ and stomatal conductance *g*_s_ for the different treatments control (C), drought (D), heat (H), heat-drought (HD) and seedlings that died (dead) during the heatwave experiment. Values are experimental phase averages of daily averages per seedling (*n*_C_=4, *n*_D_=4, *n*_H_=3, *n*_HD_=1, *n*_dead_=4) with ±1SE given.

| Experimental Phase | Treatment | Status | *E* | | | *A*_net_ | | | *g*_s_ | | |
| --- | --- | --- | --- | --- | --- | --- | --- | --- | --- | --- | --- |
|  |  |  | mmol m-2s-1 | | | µmol m-2s-1 | | | mmol m-2s-1 | | |
| Reference |  | dead | 1.03 | ± | 0.02 | 8.88 | ± | 0.18 | 53.0 | ± | 1.2 |
| Heatwave 1 |  | dead | 0.95 | ± | 0.08 | 3.62 | ± | 0.77 | 23.2 | ± | 3.8 |
| Recovery |  | dead | 0.17 | ± | 0.02 | 0.91 | ± | 0.18 | 6.2 | ± | 0.9 |
| Heatwave2 |  | dead | 0.11 | ± | 0.02 | -0.09 | ± | 0.09 | 1.5 | ± | 0.3 |
| Recovery |  | dead | 0.03 | ± | 0.01 | 0.10 | ± | 0.06 | 1.1 | ± | 0.2 |
| Rewetting |  | dead | 0.02 | ± | 0.00 | -0.04 | ± | 0.01 | 0.6 | ± | 0.1 |
| Reference | C | alive | 1.50 | ± | 0.06 | 10.63 | ± | 0.34 | 75.5 | ± | 3.7 |
| Heatwave 1 | C | alive | 1.83 | ± | 0.13 | 11.32 | ± | 0.77 | 80.4 | ± | 8.0 |
| Recovery | C | alive | 1.75 | ± | 0.12 | 10.39 | ± | 0.64 | 73.8 | ± | 6.3 |
| Heatwave2 | C | alive | 2.01 | ± | 0.19 | 10.93 | ± | 1.10 | 72.3 | ± | 8.8 |
| Recovery | C | alive | 1.41 | ± | 0.10 | 8.19 | ± | 0.55 | 55.1 | ± | 5.0 |
| Rewetting | C | alive | 1.41 | ± | 0.08 | 7.80 | ± | 0.39 | 51.7 | ± | 3.4 |
| Reference | D | alive | 1.68 | ± | 0.08 | 10.99 | ± | 0.45 | 77.2 | ± | 3.4 |
| Heatwave 1 | D | alive | 1.25 | ± | 0.19 | 8.08 | ± | 1.05 | 47.6 | ± | 6.9 |
| Recovery | D | alive | 0.83 | ± | 0.11 | 5.70 | ± | 0.74 | 30.2 | ± | 4.5 |
| Heatwave2 | D | alive | 0.86 | ± | 0.16 | 5.18 | ± | 0.92 | 25.6 | ± | 4.8 |
| Recovery | D | alive | 0.80 | ± | 0.10 | 5.09 | ± | 0.61 | 26.1 | ± | 3.3 |
| Rewetting | D | alive | 1.38 | ± | 0.14 | 7.36 | ± | 0.56 | 45.9 | ± | 4.3 |
| Reference | H | alive | 1.33 | ± | 0.07 | 11.31 | ± | 0.58 | 70.8 | ± | 3.0 |
| Heatwave 1 | H | alive | 2.51 | ± | 0.35 | 7.01 | ± | 1.25 | 52.0 | ± | 6.3 |
| Recovery | H | alive | 1.08 | ± | 0.16 | 7.14 | ± | 0.87 | 41.8 | ± | 5.7 |
| Heatwave2 | H | alive | 2.05 | ± | 0.32 | 4.28 | ± | 0.65 | 29.7 | ± | 4.4 |
| Recovery | H | alive | 1.06 | ± | 0.16 | 6.42 | ± | 0.94 | 38.6 | ± | 6.1 |
| Rewetting | H | alive | 1.30 | ± | 0.13 | 7.58 | ± | 0.74 | 43.2 | ± | 4.5 |
| Reference | HD | alive | 1.47 | ± | 0.07 | 10.61 | ± | 0.38 | 80.9 | ± | 3.7 |
| Heatwave 1 | HD | alive | 1.56 | ± | 0.23 | 5.70 | ± | 2.14 | 38.1 | ± | 13.0 |
| Recovery | HD | alive | 0.48 | ± | 0.03 | 3.98 | ± | 0.43 | 20.6 | ± | 2.8 |
| Heatwave2 | HD | alive | 0.50 | ± | 0.05 | 1.50 | ± | 0.21 | 7.9 | ± | 0.7 |
| Recovery | HD | alive | 0.34 | ± | 0.05 | 2.56 | ± | 0.38 | 11.4 | ± | 1.8 |
| Rewetting | HD | alive | 0.66 | ± | 0.04 | 4.40 | ± | 0.17 | 21.6 | ± | 1.0 |

Table S4: Shown are all continuously analyzed BVOCs for the different treatments control (C), drought (D), heat (H), heat-drought (HD) and seedlings that died (dead) during the heatwave experiment. Values are Experimental Phase averages of daily averages per seedling (*n*_C_=4, *n*_D_=4, *n*_H_=3, *n*_HD_=1, *n*_dead_=4) with ±1SE given.

| Experimental Phase | Treat | status | m33 | | | | m45 | | | m59 | | | m99 | | | m137 | | | m153 | | | |  |
| --- | --- | --- | --- | --- | --- | --- | --- | --- | --- | --- | --- | --- | --- | --- | --- | --- | --- | --- | --- | --- | --- | --- | --- |
|  |  |  | nmol m-2s-1 | | | | nmol m-2s-1 | | | nmol m-2s-1 | | | nmol m-2s-1 | | | nmol m-2s-1 | | | nmol m-2s-1 | | | |  |
| Reference |  | dead | 0.25 | ± | 0.02 | | 0.03 | ± | 0.01 | 1.30 | ± | 0.07 | 0.010 | ± | 0.001 | 0.47 | ± | 0.07 | | 0.003 | ± | 0.000 | |
| Heatwave 1 |  | dead | 0.99 | ± | 0.24 | | 0.06 | ± | 0.01 | 1.24 | ± | 0.18 | 0.025 | ± | 0.004 | 3.71 | ± | 1.13 | | 0.012 | ± | 0.002 | |
| Recovery |  | dead | 0.88 | ± | 0.24 | | 0.01 | ± | 0.00 | 0.98 | ± | 0.17 | 0.010 | ± | 0.002 | 1.57 | ± | 0.29 | | 0.003 | ± | 0.001 | |
| Heatwave2 |  | dead | 4.39 | ± | 1.24 | | 0.38 | ± | 0.15 | 1.41 | ± | 0.40 | 0.045 | ± | 0.013 | 8.32 | ± | 2.93 | | 0.022 | ± | 0.008 | |
| Recovery |  | dead | 0.36 | ± | 0.07 | | 0.13 | ± | 0.02 | 0.39 | ± | 0.06 | 0.009 | ± | 0.002 | 0.98 | ± | 0.16 | | 0.003 | ± | 0.001 | |
| Rewetting |  | dead | 0.39 | ± | 0.06 | | 0.19 | ± | 0.03 | 0.36 | ± | 0.04 | 0.010 | ± | 0.001 | 0.74 | ± | 0.16 | | 0.002 | ± | 0.001 | |
| Reference | C | alive | 0.41 | ± | 0.02 | | 0.04 | ± | 0.00 | 1.41 | ± | 0.11 | 0.014 | ± | 0.002 | 0.41 | ± | 0.06 | | 0.003 | ± | 0.000 | |
| Heatwave 1 | C | alive | 0.58 | ± | 0.05 | | 0.05 | ± | 0.01 | 1.89 | ± | 0.27 | 0.020 | ± | 0.002 | 0.38 | ± | 0.06 | | 0.005 | ± | 0.001 | |
| Recovery | C | alive | 0.58 | ± | 0.05 | | 0.04 | ± | 0.01 | 1.51 | ± | 0.21 | 0.017 | ± | 0.002 | 0.31 | ± | 0.05 | | 0.005 | ± | 0.001 | |
| Heatwave2 | C | alive | 0.81 | ± | 0.07 | | 0.07 | ± | 0.01 | 1.56 | ± | 0.34 | 0.025 | ± | 0.003 | 0.49 | ± | 0.07 | | 0.006 | ± | 0.001 | |
| Recovery | C | alive | 0.55 | ± | 0.05 | | 0.05 | ± | 0.01 | 1.27 | ± | 0.17 | 0.012 | ± | 0.001 | 0.39 | ± | 0.08 | | 0.005 | ± | 0.001 | |
| Rewetting | C | alive | 0.56 | ± | 0.04 | | 0.06 | ± | 0.01 | 1.27 | ± | 0.15 | 0.017 | ± | 0.001 | 0.71 | ± | 0.08 | | 0.006 | ± | 0.001 | |
| Reference | D | alive | 0.48 | ± | 0.03 | | 0.04 | ± | 0.00 | 1.83 | ± | 0.16 | 0.019 | ± | 0.002 | 0.70 | ± | 0.12 | | 0.004 | ± | 0.000 | |
| Heatwave 1 | D | alive | 0.47 | ± | 0.08 | | 0.04 | ± | 0.01 | 1.47 | ± | 0.22 | 0.016 | ± | 0.004 | 0.58 | ± | 0.08 | | 0.003 | ± | 0.000 | |
| Recovery | D | alive | 0.27 | ± | 0.05 | | 0.03 | ± | 0.01 | 0.49 | ± | 0.07 | 0.011 | ± | 0.004 | 0.27 | ± | 0.04 | | 0.003 | ± | 0.000 | |
| Heatwave2 | D | alive | 0.37 | ± | 0.10 | | 0.05 | ± | 0.02 | 0.41 | ± | 0.09 | 0.011 | ± | 0.003 | 0.32 | ± | 0.06 | | 0.002 | ± | 0.000 | |
| Recovery | D | alive | 0.27 | ± | 0.04 | | 0.04 | ± | 0.01 | 0.45 | ± | 0.08 | 0.007 | ± | 0.002 | 0.23 | ± | 0.04 | | 0.003 | ± | 0.001 | |
| Rewetting | D | alive | 0.53 | ± | 0.06 | | 0.03 | ± | 0.00 | 1.01 | ± | 0.10 | 0.010 | ± | 0.002 | 0.42 | ± | 0.04 | | 0.006 | ± | 0.001 | |
| Reference | H | alive | 0.29 | ± | 0.02 | | 0.03 | ± | 0.01 | 1.61 | ± | 0.15 | 0.012 | ± | 0.002 | 1.28 | ± | 0.17 | | 0.004 | ± | 0.001 | |
| Heatwave 1 | H | alive | 1.56 | ± | 0.41 | | 0.10 | ± | 0.03 | 2.27 | ± | 0.37 | 0.028 | ± | 0.009 | 4.33 | ± | 1.16 | | 0.019 | ± | 0.005 | |
| Recovery | H | alive | 0.43 | ± | 0.08 | | 0.01 | ± | 0.01 | 0.87 | ± | 0.18 | 0.013 | ± | 0.003 | 1.01 | ± | 0.24 | | 0.002 | ± | 0.000 | |
| Heatwave2 | H | alive | 1.63 | ± | 0.28 | | 0.13 | ± | 0.04 | 1.50 | ± | 0.28 | 0.031 | ± | 0.008 | 2.35 | ± | 0.46 | | 0.013 | ± | 0.003 | |
| Recovery | H | alive | 0.33 | ± | 0.04 | | 0.03 | ± | 0.01 | 1.23 | ± | 0.17 | 0.006 | ± | 0.001 | 0.31 | ± | 0.04 | | 0.002 | ± | 0.000 | |
| Rewetting | H | alive | 0.35 | ± | 0.04 | | 0.03 | ± | 0.01 | 1.65 | ± | 0.17 | 0.008 | ± | 0.002 | 0.53 | ± | 0.06 | | 0.002 | ± | 0.000 | |
| Reference | HD | alive | 0.33 | ± | 0.02 | | 0.01 | ± | 0.01 | 1.07 | ± | 0.06 | 0.007 | ± | 0.003 | 1.03 | ± | 0.12 | | 0.004 | ± | 0.001 | |
| Heatwave 1 | HD | alive | 0.93 | ± | 0.25 | | 0.05 | ± | 0.01 | 1.62 | ± | 0.34 | 0.023 | ± | 0.006 | 4.32 | ± | 1.00 | | 0.013 | ± | 0.003 | |
| Recovery | HD | alive | 0.15 | ± | 0.06 | -0.02 | | ± | 0.01 | 0.27 | ± | 0.03 | -0.001 | ± | 0.005 | 0.39 | ± | 0.06 | | 0.000 | ± | 0.001 | |
| Heatwave2 | HD | alive | 0.93 | ± | 0.57 | | 0.15 | ± | 0.17 | 0.55 | ± | 0.06 | 0.031 | ± | 0.025 | 1.14 | ± | 0.16 | | 0.001 | ± | 0.001 | |
| Recovery | HD | alive | 0.08 | ± | 0.01 | | 0.01 | ± | 0.01 | 0.20 | ± | 0.04 | 0.002 | ± | 0.004 | 0.07 | ± | 0.01 | | 0.000 | ± | 0.000 | |
| Rewetting | HD | alive | 0.21 | ± | 0.03 | | 0.01 | ± | 0.01 | 0.68 | ± | 0.06 | 0.000 | ± | 0.003 | 0.20 | ± | 0.04 | | 0.001 | ± | 0.000 | |

Table S5 Shoot biomass, leaf area (half-sided) and monoterpene (MT) pools and emissions from the surviving heat-treated (H, HD) and control (C) seedlings measured in the gas exchange chambers. Cumulative MT emissions are shown from the first day of heatwave 1 to the last day of heatwave 2. Endogenous MT pools were scaled to the seedling-level using the median of the MT pool per heat and control treatment multiplied by the biomass per seedlings. Note that seedlings in the cuvettes and seedlings analyzed for MT pools were not the same because whole seedlings where sampled – Biomass of the cuvette seedlings (shoot [g]) was taken at the end of the experiment.

| Treatment | Cuvette num. | Shoot  [g] | MT pool  [mg] | MT Emission  [mg] | Emission % from total pool |
| --- | --- | --- | --- | --- | --- |
| H | 8 | 16.02 | 189 | 34.18 | 18.1 |
| H | 11 | 9.65 | 114 | 25.72 | 22.5 |
| H | 16 | 17.53 | 207 | 35.94 | 17.4 |
| HD | 12 | 6.51 | 80 | 16.67 | 20.8 |
| C | 2 | 21.79 | 284 | 13.78 | 4.9 |
| C | 5 | 13.58 | 177 | 10.01 | 5.6 |
| C | 10 | 9.05 | 118 | 2.13 | 1.8 |
| C | 13 | 8.61 | 112 | 4.72 | 4.2 |

Figure S1


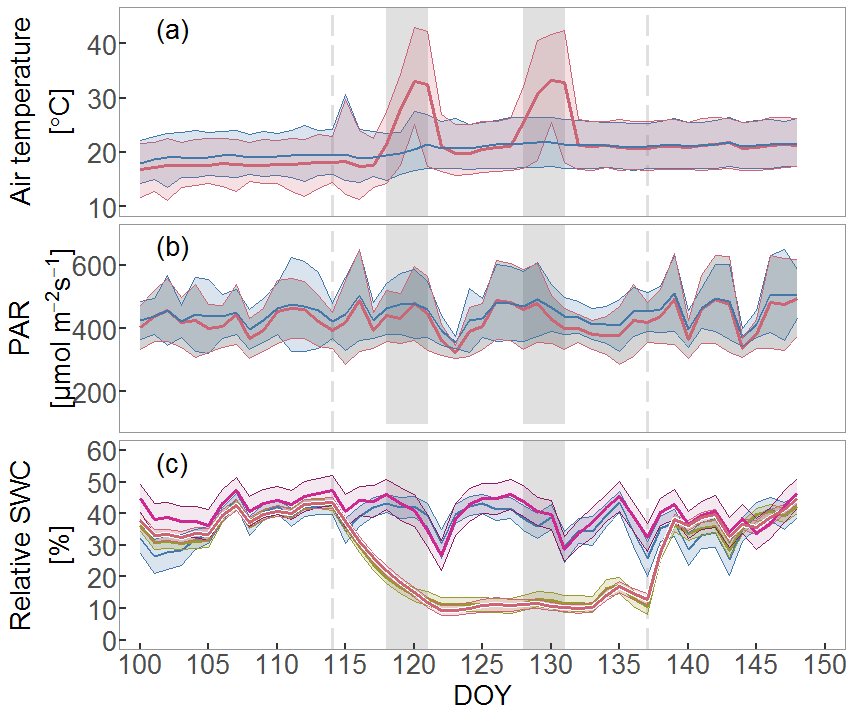


Figure S1: Overview of the environmental drivers during the heatwave experiment. Shown are air temperature (a) and photosynthetic active radiation PAR (b) of the heat (red) and non-heat (blue) treatments. Relative substrate water content rSWC (c) is shown for control (blue), drought (green), heat (magenta) and heat-drought (red) with *n*=10 per treatment. Data are shown as daily averages and standard deviation (±1SD) for (a,c) and for the 16 hours light period in (b). This is a modified figure previously published in (Birami et al. 2018).

Figure S2


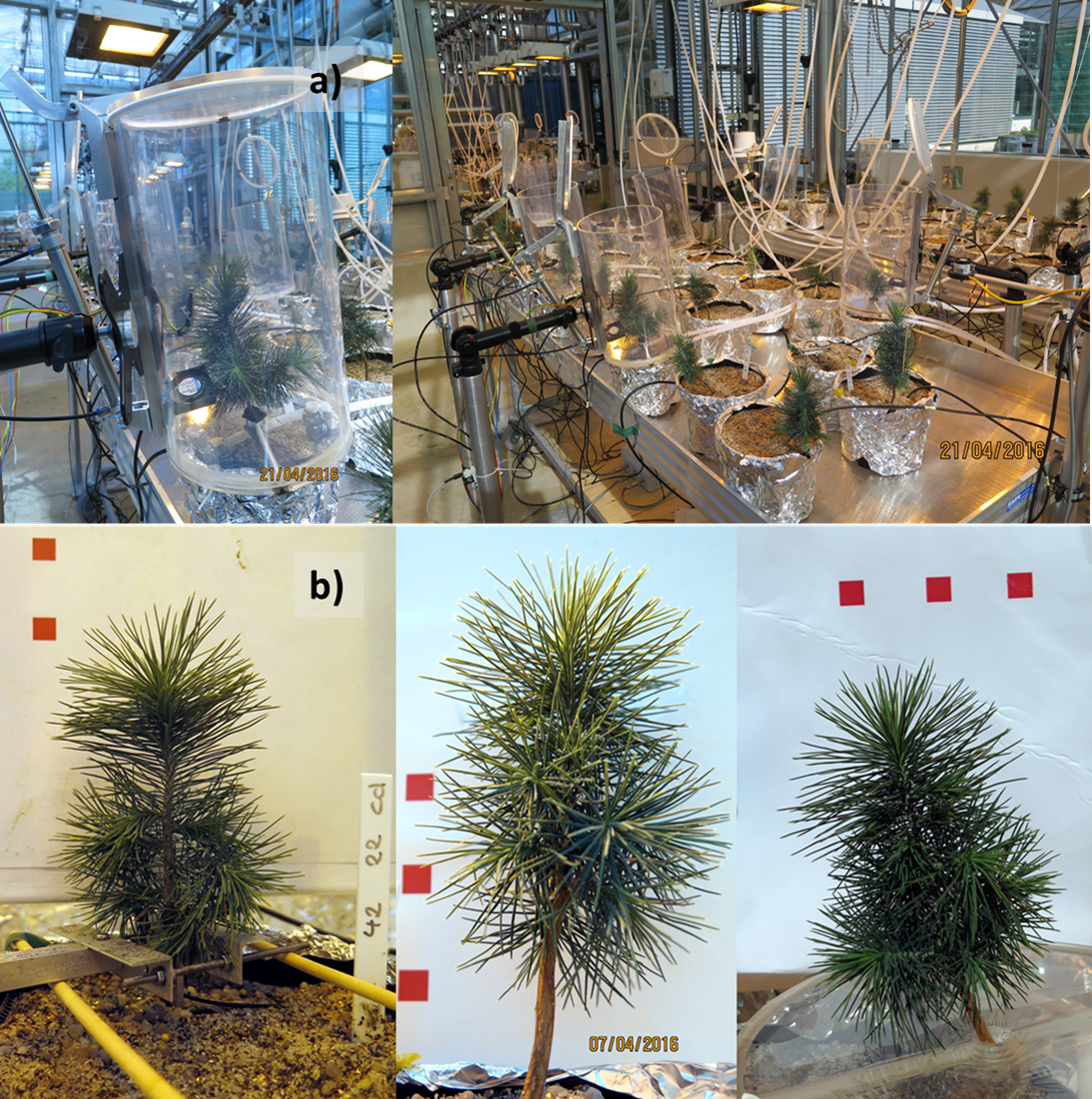


Figure S2: Experimental setup together with a gas exchange cuvette placement example (a), and example photographs, which were taken from seedlings to derive their projected leaf area (b). Red squares are 1 cm² each to calibrate for the estimated needle area. Area was estimated using the software ImageJ, color threshold was adjusted with method “RemyiEntropy”,RED, HSB, Hue:60/113,Saturation:40/255, Brightness: 35/130.

Figure S3


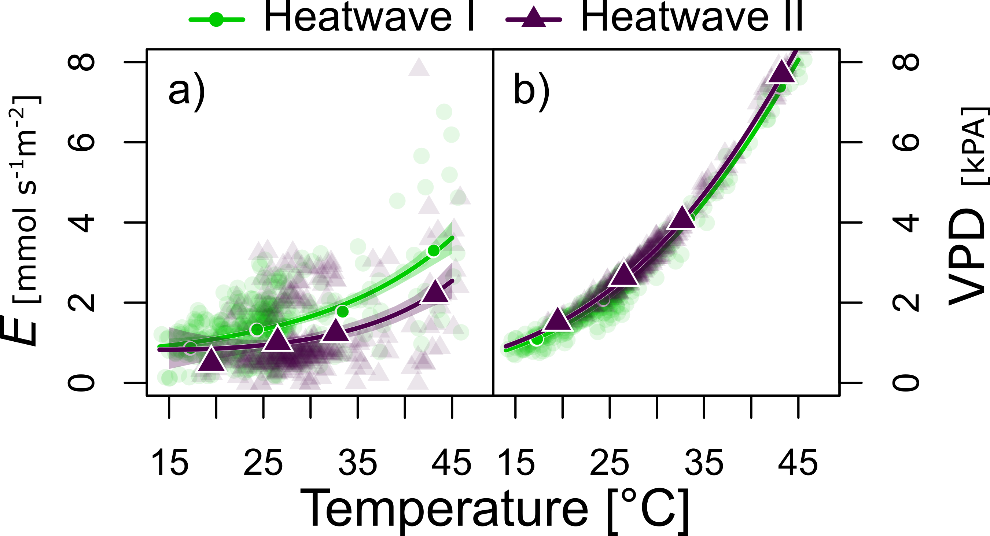


Figure S3: Differences in temperature responses of Transpiration (E,a)) and vapor pressure deficit (VPD, b)) between the first (filled circles, light green) and second heatwave (triangles, lilac) of Aleppo pine seedlings is depicted by exponential functions (exp(b(T)+c). Data are measurements of the surviving heat treated seedlings (n=3) for PAR ≥ 100 µmol m^-2^s^-1^ including several days before each heatwave to investigate a larger temperature range. Shaded areas depict the 95% confidence intervals of the fitted functions. Larger shapes depict bin-averaged data. Note that VPD increased exponentially with temperature: VPD=exp(b(T)+c, with b= 0.066[0.0658-0.0669 CI95%] and c=-0.85[-0.08678 - -0.0829 CI95%], R^2^ = 0.94 of log-transformed function.

Figure S4


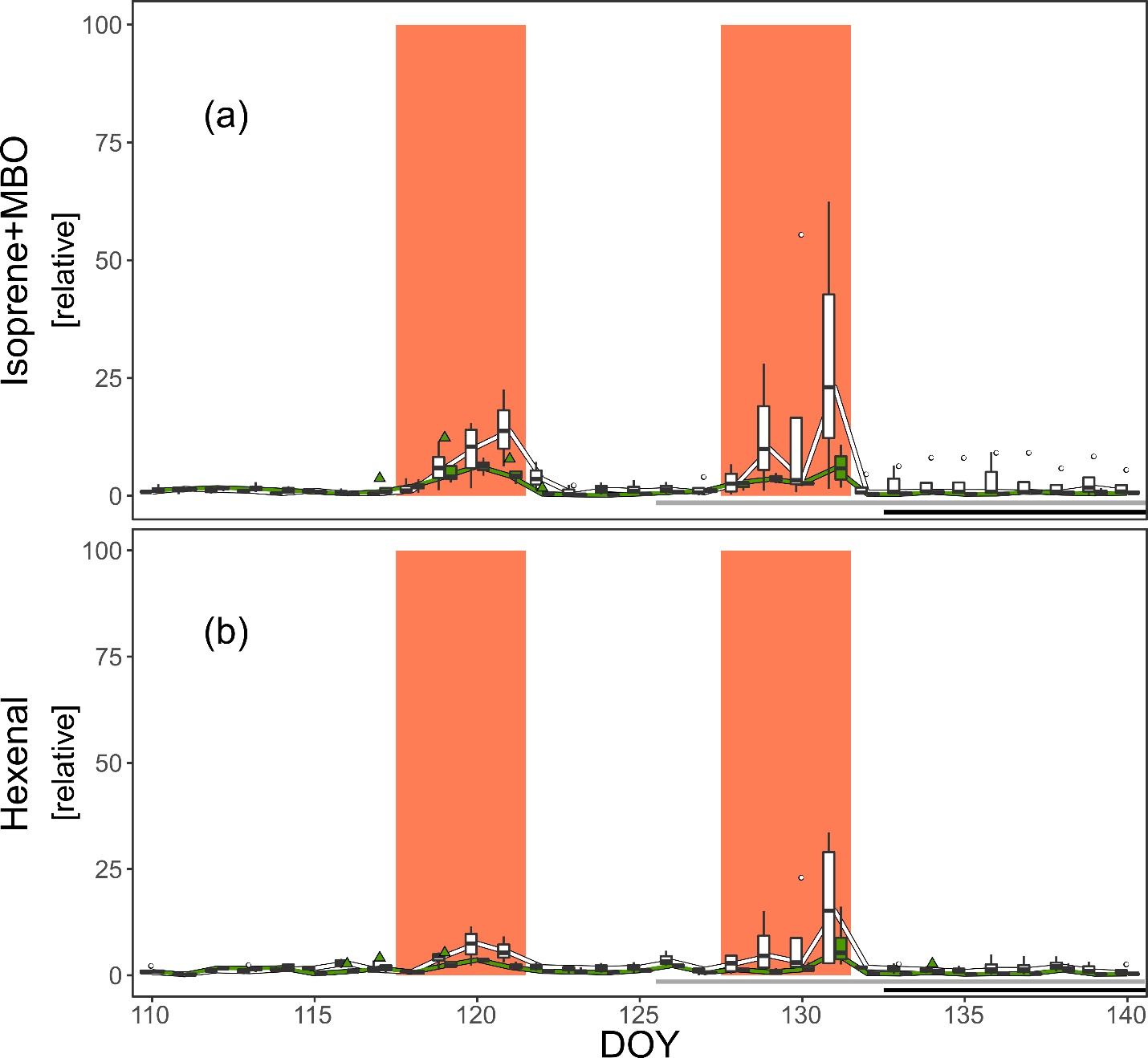


Figure S4 Responses of Isoprene + MBO (a) and hexenal (b) emissions during tree mortality. The data is presented as boxplots derived from daily-averages per seedling, separated in surviving (green, *n*=4) and dying (white, *n*=4). The two heatwaves are highlighted by a solid colored background (DOY 118-121; 128-131 Horizontal grey bars mark the time course on when daily-averaged transpiration and net photosynthesis of the dying seedlings reached zero. Dark respiration ceased is indicated by black horizontal bars (asymptotic approximation to 0). Data shown originate from the seedlings of the heat and heat-drought treatment. Daily-averages of emission data is for PAR ≥ 100

References

Birami B, Gattmann M, Heyer AG, Grote R, Arneth A, Ruehr NK (2018) Heat Waves Alter Carbon Allocation and Increase Mortality of Aleppo Pine Under Dry Conditions. Front. For. Glob. Change 1:1285. https://doi.org/10.3389/ffgc.2018.00008
